# Isolation and Purification of Bioactive Compounds from the Stem Bark of *Jatropha podagrica*

**DOI:** 10.3390/molecules24050889

**Published:** 2019-03-03

**Authors:** Truong Ngoc Minh, Tran Dang Xuan, Hoang-Dung Tran, Truong Mai Van, Yusuf Andriana, Tran Dang Khanh, Nguyen Van Quan, Ateeque Ahmad

**Affiliations:** 1 Graduate School for International Development and Cooperation (IDEC), Hiroshima University, Higashi-Hiroshima 739-8529, Japan; minhtn689@gmail.com (T.N.M.); truongmaivan1991@gmail.com (T.M.V.); yusufandriana@yahoo.com (Y.A.); nguyenquan26@gmail.com (N.V.Q.); 2 Department of Biotechnology, NTT Institute of Hi-Technology, Nguyen-Tat-Thanh University, 298A-300A Nguyen-Tat-Thanh Street, District 04, Ho chi Minh City 72820, Vietnam; 3 Agricultural Genetics Institute, Hanoi City 123000, Vietnam; tdkhanh@vaas.vn; 4 Center for Expert, Vietnam National University of Agriculture, Hanoi 131000, Vietnam; 5 Chemical Engineering, CSIR, CIMAP, Kukrail Picnic Spot Road, Lucknow 226016, India; ateeque97@gmail.com

**Keywords:** *Jatropha podagrica*, stem bark, gallic acid, fraxetin, methyl gallate, tomentin, antioxidant, antibacterial, allelopathic activity

## Abstract

This paper reports the successive isolation and purification of bioactive compounds from the stem bark of *Jatropha podagrica*, a widely known medicinal plant. The ethyl acetate extract of the stem bark exhibited the strongest antioxidant activity assessed by 2,2-diphenyl-1-picrylhydrazyl (DPPH), 2,2′-azinobis-(3-ethylbenzothiazoline-6-sulfonic acid) (ABTS) radical scavenging, and ferric reducing antioxidant power (FRAP) assays (IC_50_ = 46.7, 66.0, and 492.6, respectively). By column chromatography (CC) with elution of hexane and ethyl acetate at 8:2, 7:3, and 6:4 ratios, the isolation of this active extract yielded five fractions (**C1**–**C5**). Chemical structures of the constituents included in **C1**–**C5** were elucidated by gas chromatography-mass spectrometry (GC-MS), electrospray ionization-mass spectrometry (ESI-MS), and nuclear magnetic resonance (NMR) and resolved as methyl gallate (**C1**, **C2**, **C3**, **C4**), gallic acid (**C1**, **C2**), fraxetin (**C2**, **C3**, **C4**, **C5**), and tomentin (**C3**). Mixture **C2** (IC_50_ DPPH and ABTS = 2.5 µg/mL) and **C3** (IC_50_ FRAP = 381 µg/mL) showed the highest antioxidant properties. Among the isolated fractions, **C4** was the most potential agent in growth inhibition of six bacterial strains including *Staphylococcus aureus*, *Escherichia coli*, *Klebsiella pneumoniae, Listeria monocytogenes,* *Bacillus subtilis*, and *Proteus mirabilis* (MIC = 5, 20, 30, 20, 25, and 20 mg/mL, respectively). All identified constituents exerted an inhibitory activity on the growth of *Lactuca sativa*, of which the mixture **C3** performed the maximal inhibition on shoot (IC_50_ = 49.4 µg/mL) and root (IC_50_ = 47.1 µg/mL) growth. Findings of this study suggest that gallic acid, methyl gallate, fraxetin, and tomentin isolated from *J. podagrica* possessed antioxidant, antibacterial, and growth inhibitory potentials.

## 1. Introduction

Plants synthesize numerous natural organic compounds having complex chemical structures. These plant-derived compounds play a crucial role in their ecological functions [1]. Extensive studies have indicated that terpenoids, phenolics, and nitrogen-containing substances are important phytoalexins which provide a defense system and protect plants against attack by harmful microbes and herbivores [1,2]. The plants that release these active compounds are capable to compete and invade other plant species in their vicinity by suppressing their growth (a natural phenomenon known as allelopathy) [3,4]. In addition to their physiological functions in plants, numerous phytoalexins have also been reported to possess strong antioxidant, antibacterial, and herbicidal properties. A number of bioactive compounds have been isolated, purified and employed in a wide range of applications including food, pharmaceutical, cosmetic, and agricultural industries [1,5,6,7]. Therefore, the exploration of active medicinal plants and their natural bioactive molecules has become essential to exploit the possible additional values of natural sources.

*Jatropha podagrica* is a succulent shrub belonging to the family Euphorbiaceae. It is widely distributed in tropical and subtropical areas worldwide [8]. *J. podagrica* is an ornamental plant and one of the most important materials of traditional folklore medicines in Asia, Latin America, and Africa [9,10,11]. In traditional therapies, this plant has been extensively used as an effective treatment for skin infections [8], jaundice and fever [12], sexually transmitted diseases like gonorrhea [8,13] pain relief [14], gout [15], and paralysis [15,16]. In addition, its seed oil is applied in African ethnomedical practice as a natural remedy for rheumatic conditions, pruritus, and to alleviate constipation, while its leaves have been used as a hemostatic agent (IPCS-INCHEM). In Nigeria, indigenous people utilize this shrub to cure hepatitis [17]. The investigations of the biologically active components of *J. podagrica* resulted in the isolations of japodic acid, erytrinasinate, fraxidin [13], steroids and flavonoids [18], podacycline A and B [19], diterpenoids, japodagrone, japodagrin [8], 3-acetylaleuritolic acid, japodagrol [20], n-heptyl ferulate, and γ-sitosterol [21]. Although extracts of different parts of *J. podagrica* have been reported to possess various biological properties including antiproliferative, antioxidant, antitumor, antibacterial, and antimicrobial [8,12,13], the search for phytochemicals responsible for the observed activities has been conducted sporadically, except for antibacterial capacity. Additionally, little information has been found concerning phytochemicals and biological activity from *J. podagrica* stem bark so far [22]. Several second metabolites including fraxidin, fraxetin, scoparone, 3-acetylaleuritolic acid, β-sitosterol, and sitosterone from the stem bark of this plant were isolated, but their biological activity was not examined [21]. In another trial, although the antimicrobial activity of *J. podagrica* stem bark extracts was evaluated, isolation of the compounds responsible for the studied activity was not achieved. Therefore, the objectives of this research were to establish a simple and effective protocol to isolate the bioactive components present in *J. podagrica* stem bark. The bioactive properties including antioxidant, antibacterial, and plant growth inhibitory activities were also evaluated.

## 2. Results

### 2.1. Antioxidant Activities of the Different Fractions of J. podagrica Stem Bark Extract

**Figure 1:**
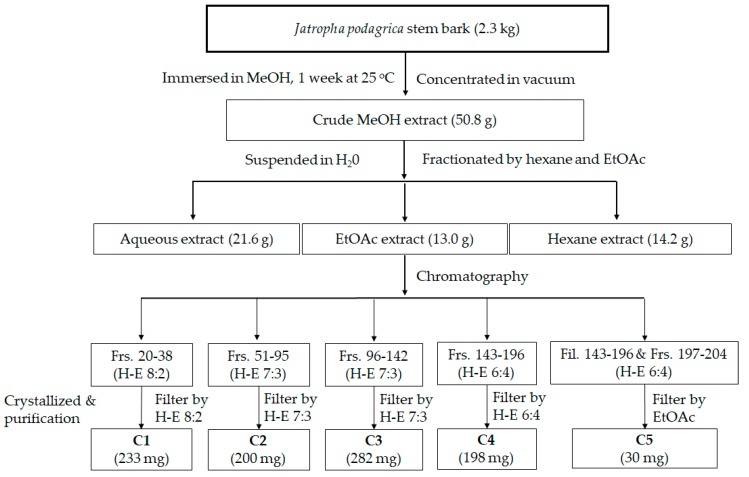
Procedure of extraction, fractionation, and isolation of bioactive compounds from *Jatropha podagrica* stem bark (H: Hexane, E: Ethyl acetate, Frs.: Fractions, Fil.: Filtration). The fractions are labelled **C1**–**C5**.

Chromatographic fractionation of the crude methanol extract led to three different polarity extracts including hexane, EtOAc, and aqueous. The DPPH, ABTS radical scavenging activities, and ferric reducing antioxidant power (FRAP) of individual extracts were then performed to evaluate the antioxidant capacities of the extract fractions. Among them, EtOAc emerged as the most effective solvent with the lowest IC_50_ values in scavenging both DPPH and ABTS free radicals and reducing ferric power. Its IC_50_ values in the three assays were 46.7, 66.0, and 492.6 µg/mL, respectively. The results imply that the antioxidant constituents originating from *J. podagrica* stem bark might predominantly be present in the EtOAc extract. Hence, this extract was selected for further isolation and purification of the bioactive compounds responsible for antioxidant activity of this shrub succulent plant. The bioactive constituents of the ethyl acetate extract of *J. podagrica* stem bark were isolated using column chromatography (Figure 1, Table 1).

### 2.2. Structure Elucidation of Isolated Compounds

Separation of bioactive compounds from *J. podagrica* was conducted following the procedure illustrated in Figure 1. The methanol extract of this plant (50.8 g) was extracted with hexane, ethyl acetate, and water to produce 14.2, 13.0, and 21.6 extracts, respectively. The ethyl acetate extract was then separated by column chromatography using a gradient elution technique as follows: Fractions (Frs.) 20–38 (hexane: EtOAc = 8:2), Frs. 51–95 (hexane: EtOAc = 7:3), Frs. 96–142 (hexane: EtOAc = 7:3), Frs. 143–196 (hexane: EtOAc = 6:4) to obtain 223 mg (**C1**), 200 mg (**C2**), 282 mg (**C3**), and 228 mg (**C4**) of compounds. **C5** (30 mg) was the mixture Frs. 197–204 and residue of Frs. 143–196 after filtration by EtOAc.

**Figure 2:**
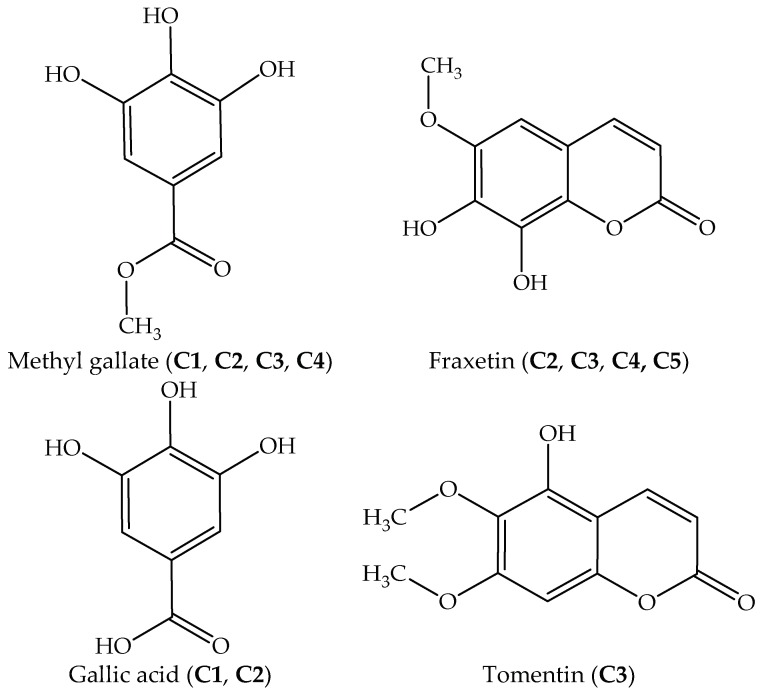
Chemical structures of bioactive constituents identified in EtOAc extract of *Jatropha podagrica* stem bark.

On the basic of GC-MS data, the retention times, molecular weight, molecular formula, name of compound, similarity, and peak area (%) of components in each isolated fraction were identified (Table 2). The structure and formula of the compounds were further confirmed by ESI-MS and LC-MS. Only fraction **C5** was additionally elucidated by ^1^H-NMR and ^13^C-NMR. The instrument analyses including GC-MS, ESI-MS, and LC-MS to elucidate the chemical structures of (1) methyl gallate: [C_8_H_8_O_5_ – H]^−^: 183.02-183.30; [C_8_H_8_O_5_ + H]^+^: 184.02; [C_8_H_8_O_5_ + Na]^+^: 207.03 (**C1**, **C2**, **C3**, and **C4**) major fragments: 184.08, 154.06, 153.05, 125.05, 79.04, (2) fraxetin: [C_10_H_8_O_5_ – H]^−^: 207.02-207.23; [C_10_H_8_O_5_ + H]^+^: 209.04; [C_10_H_8_O_5_ + Na]^+^: 231.02; major fragments: 209.09, 208.09, 193.06, 180.09, 165.06, 137.06, 109.05, 81.05, 53.05, 51.04 (**C2**, **C3**, **C4**, and **C5**), (3) gallic acid: [C_7_H_6_O_5_ – H]^−^: 169.01-169.27; [C_7_H_6_O_5_ + H]^+^: 171.04; [C_7_H_6_O_5_ + Na]^+^: 193.02; major fragments: 170.12, 152.02, 124.01, 79.04 (**C1**, **C2**), and (4) tomentin: [C_11_H_10_O_5_ – H]^−^: 221.04; [C_11_H_10_O_5_ + H]^+^: 223.05-223.06; [C_11_H_10_O_5_ + Na]^+^: 245.04; major fragments: 222.05, 221.04, 207.02, 194.05, 178.06, 163.03, 152.04, 136.01, 108.02, 54.01, 41.00 (**C4**) (Supplementary Materials S1–S21). Chemical structures of the identified compounds are illustrated in Figure 2.

### 2.3. Quantitative Analysis of Fraxetin from J. podagrica Stem Bark

The content of one pure compound fraxetin in the stem bark of *J. podagrica* is presented in Table 3. Quantification of this compound was done by GC (13.04 µg/g DW—dry weight) by comparing the mass spectra, retention time, and peak area between the samples and purified fraxetin from this research.

### 2.4. Antioxidant Activities of the Isolated Fractions

Antioxidant activities of the isolated fractions were determined using three assays including DPPH, ABTS free radical scavenging, and reducing power assay. BHT was used as a standard for all methods. The results are summarized in Table 4.

#### 2.4.1. DPPH Activity of the Isolated Fractions

As can be seen in Table 4, the DPPH scavenging activities of **C2** was the strongest as its IC_50_ value was the lowest (2.5 µg/mL). Statistically, the antioxidant activities of **C4** and **C5** (IC_50_ = 3.54 and 2.81 µg/mL, respectively) were similar in comparison with that of **C2**. The fraction **C3** (IC_50_ = 6.92 µg/mL) exhibited intermediate antioxidant capacities, while **C1** showed the lowest DPPH radical scavenging ability (IC_50_ = 40.83 µg/mL). Generally, the antioxidant properties of the isolated fractions were higher than that displayed by BHT, except for the fraction **C1**.

#### 2.4.2. ABTS Activity of the Isolated Fractions

The ABTS scavenging abilities of the fractions and BHT are presented in Table 4. Among the tested samples, **C2** exhibited the maximum scavenging activity with an IC_50_ value of 2.5 µg/mL, followed by **C5**, **C4**, and **C3** although their IC_50_ values were not significantly different (IC_50_ = 3.23, 3.36, and 7.52 µg/mL). The lowest antioxidant property was found for fraction **C1** (IC_50_ = 21.83 µg/mL). This result is consistent with that of the DPPH method. However, all tested fractions showed stronger ABTS scavenging abilities as compared with that of the positive control BHT.

#### 2.4.3. Ferric Reducing Antioxidant Activity of the Isolated Fractions

The reducing power ability of various fractions and BHT are summarized in Table 4. Among the isolated fractions, **C5** displayed the maximum reducing power with an IC_50_ value of 381 µg/mL. The antioxidant activity of the tested samples evaluated by the FRAP method could be ranked in the following order: **C5** > **C4** > **C2** > **C1** > **C3** > BHT.

### 2.5. Antibacterial Activity of the Isolated Fractions

The antibacterial activities of the different fractions of the EtOAc extract of *J. podagrica* against the growth of six bacteria including Gram-positive bacteria: *Staphylococcus aureus, Listeria monocytogenes*, *Bacillus subtilis* and Gram-negative bacteria: *Escherichia coli*, *Klebsiella pneumoniae*, *Proteus mirabilis* are shown in Table 5. Ampicillin and streptomycin were used as standards. The results revealed that all the fractions are potential antibacterial agents against most of the investigated microorganisms. However, the inhibitory effects varied among the bacteria and tested samples.

It was found that fractions **C3**, **C4**, and **C5** inhibited all tested pathogens, but **C1** was inactive on *K. pneumonia* and *P. mirabilis*, and **C2** also had no effect on the growth of *K. pneumoniae*. Among the studied fractions, **C4** exerted the strongest inhibitory effects on the growth of four bacterial strains including *S. aureus*, *E. coli*, *B. subtilis*, *L. monocytogenes*, and *P. mirabilis*. The MIC values of **C4** were 5, 20, 20, 25, and 20 mg/mL, respectively, whilst *K. pneumoniae* was the most sensitive to the fraction **C5** with the MIC 25 mg/mL.

### 2.6. Growth Inhibitory Activities of the Isolated Fractions

The allelopathic capacities of the isolated fractions on the growth of *Raphanus sativus, Echinochloa crus-galli,* and *Lactuca sativa* are presented in Table 6. The herbicidal activities of different fractions were also compared, expressed by the IC_50_ value, which presented the concentration required to inhibit 50% growth of the indicator plants. The growth inhibitory effects on the tested plants performed differently with the isolated fractions and compounds of *J. podagrica* bark. In general, all studied fractions from *J. podagrica* exhibited suppressive effects on the growth of tested plants and the inhibitory effects of the fractions on root lengths were higher than that of the shoot elongations. However, the emergence of *R. sativus* and *E. crus-galli* was less influenced than that of *L. sativa*. The fraction **C3** had the greatest phytotoxic effect shoot height of *L. sativa* (IC_50_ = 49.4 µg/mL), while its root was most sensitive to the fraction **C5** (IC_50_ = 30.0 µg/mL).

The fraction **C1** was the most inhibitory against the magnitude of the shoot of *E. crus-galli* (IC_50_ = 495.7 µg/mL), but **C3** exhibited the strongest inhibitory effect on its root (IC_50_ = 143.9 µg/mL). The fraction **C2** gave maximum inhibitory effects on shoot and root elongation of *R. sativus* (IC_50_ = 924.7 and 212.3 µg/mL, respectively).

## 3. Discussion

The radical scavenging activity of the isolated fractions was examined using DPPH, which is a frequently used method in natural product antioxidant evaluation [24]. Previous reports showed that methanol and water extracts of *J. podagrica* leaves and seeds exhibited antioxidant activities. By using this method, their antioxidant capacities were evaluated at IC_50_ values of 78.19 and 71.34 µg/mL, respectively [12]. In the genus *Jatropha*, the antioxidant capacity of bark extracts has been investigated for *J. curcas* [25]. At a concentration of 1000 µg/mL, the percentages of DPPH and ABTS radical scavenging activity of different extracts were shown as follows: the methanolic extract (91.5% and 89%, respectively), the aqueous extract (80.5% and 86.8%, respectively), and the ethanolic extract (78.2% and 87.78%, respectively). However, the IC_50_ values of the antioxidant activity of these extracts were not mentioned. In this study, for the first time, we found that the *J. podagrica* stem bark extracts possessed remarkable antioxidant capacity. At a concentration of 500 µg/mL, the inhibition percentage of hexane, EtOAc, and aqueous was >95% in both the DPPH and ABTS methods. The oxidation process is involved with multiple reaction characteristics and different mechanisms; hence, no single method can accurately evaluate the antioxidants in complex botanical extracts. The result indicated that this compound could easily donate an electron to Fe (III) most effectively, thus reducing it to Fe (II) [24,26]. Therefore, three different assays including DPPH, ABTS radical scavenging activities, and reducing power assay were employed to measure the antioxidant activity of various extracts of *J. podagrica*. The result obtained from these methods showed consistently that the EtOAc extract had the strongest antioxidant ability (Table 1), indicating that the antioxidants of this sub-woody shrub have been effectively enriched in this extraction. Compared with *J. podagrica* leaves and seeds, the extract of stem bark demonstrated a stronger DPPH radical scavenging activity (IC_50_ = 46.7 µg/mL, Table 1). Moreover, the antioxidant capacity of *J. podagrica* seed extract has been reported to be greater than that of ascorbic acid [12]. Our results suggest that the EtOAc extract of stem bark might contain the major antioxidants of *J. podagrica*.

Due to the considerable therapeutic values, intensive investigations of *J. podagrica* have documented the presence of many classes of plant secondary metabolites such as diterpenoids, flavonoids, steroids, cyclic peptides [8,13,18,19,20] (Table 7). However, they have primarily focused thus far on the root, leaf, and seed of this plant, while studies on stem bark have been desultory. In this study, four compounds were isolated and identified from the most effective EtOAc extract of stem bark, namely fraxetin, gallic acid, methyl gallate, and tomentin. They are biologically active constituents belonging to coumarin and phenolic acids. The antioxidant capacities of a purified compound (**C5**) and the mixtures **C1**, **C2**, **C3**, and **C4** were significantly stronger than the EtOAc extract and the standard (BHT), except for the DPPH radical scavenging activity of **C1** (methyl gallate, gallic acid) which was lower than that of BHT. Specifically, the antioxidant abilities of a mixture **C2** (fraxetin, gallic acid, and methyl gallate) and a pure compound **C5** (fraxetin) measured by DPPH and ABTS assays, were significantly higher than BHT, approximately 3 to 18 folds, respectively [27,28,29,30,31]. Fraxetin was successfully isolated from *Fraxinus rhinchophylla* and reported to have potential anti-oxidative effects [32], while gallic acid and methyl gallate have been separated from *Givotia rottleriformis* and reported to have anti-proliferative effects [33].

In addition to antioxidant potential, isolated constituents of *J. podagrica* showed considerable herbicide activity and antibacterial property. Although the antibacterial capacity of extracts and isolated compounds of *J. podagrica* has been investigated so far [8], the growth inhibitory activity of these plants was acknowledged for the first time in this study. According to the allelopathic assay, all fractions inhibited the growth of the studied plants at various levels. Generally, the level of inhibition on root lengths was higher than on shoot elongations in all plants (Table 6). The reduction in root growth might be due to the sensitivity of roots to allelochemicals. The strongest inhibitory effect was observed in the growth reduction of *L. sativa*. The results suggest that these fractions isolated from *J. podagrica* might be potent candidates for the development of novel herbicides. Allelochemicals other than the identified components of *J. podagrica* in this study and their interference mechanisms need further elaboration.

Dilution is one of the most appropriate techniques for determining the MIC value [34]. By using this method, we can estimate the lowest concentration of antimicrobial agents that will inhibit the visible growth of a microorganism. Antibacterial agents of *J. podagrica* have been reported thus far including fraxidin, fraxetin, erythrinasinate, japodgrin, japodagrone, 4z-jatrogrosidentadion, 15-epi-4z–jatrogrossidentadion, 2-hydroxyisojatrogrossidion, and 2-epihydroxyisojatrogrossidion. Those compounds exerted antibacterial activity toward *S. aureus* and *B. subtilis* with inhibition zones ranging from 12 to 35 mm at a concentration of 20 µg/disk (Table 7). However, other pathogenic bacteria such as *E. coli* and *Pseudomonas aeruginosa* were not sensitive to these compounds at the same concentration. In this study, the antibacterial effects of five isolated fractions were evaluated against the growth of six different pathogens including *S. aureus, E. coli, K. pneumoniae, L. monocytogenes, B. subtilis,* and *P. mirabilis*. The studied microorganisms are ubiquitous bacteria causing an array of serious nosocomial infections worldwide. The result revealed that most of the bacteria strains were susceptible to the fractions at the tested concentrations. Among the isolated fractions, a combination of fraxetin and methyl gallate in the mixture **C4** caused the strongest antibacterial effect on *S. aureus, E. coli, L. monocytogenes, B. subtilis,* and *P. mirabilis* (Table 5). Therefore, the reaction mechanism of this synergic effect should be further investigated. It was observed that gram-negative bacteria *K. pneumoniae* and *P. mirabilis* exhibited high resistance to treatment by **C1** and **C2**. A possible explanation for this might be that the outer membrane of gram-negative bacteria acts as a barrier which is able to protect them against the penetration of compounds, and the periplasmic space carries enzymes which can break down foreign molecules introduced from outside [35].

In this study, phytochemical investigation of stem bark of *J. podagrica* led to identification of four constituents. Among the isolated compounds, fraxetin (**C5**) was previously identified from *J. podagrica* by Rumzhum et al. [21] and has been known as an antibacterial and antidiabetic agent [36,37]. Other constituents including gallic acid, methyl gallate, and tomentin were isolated for the first time in the stem bark of *J. podagrica* herein. The content of pure fraxetin (**C5**) was quantified as 13.04 µg/g DW (Table 3). Gallic acid and methyl gallate have been documented as medicinally important components found in most plants and possess a wide range of pharmacological activities such as antioxidant, anticancer, anti-HIV, antiulcerogenic, anti-inflammatory, and antifungal [38,39]. Tomentin was firstly identified in the root of *J. curcas* which is another member of the Euphorbiaceae family [40]. The biological activities of this substance have been described as a potent anti-inflammatory capacity [41]. The result in Figure 1 suggests that the combination of hexane and ethyl acetate at 8:2, 7:3, and 6:4 was the most efficient elution to yield bioactive components from *J. podagrica* stem bark. This is the first report of methyl gallate, gallic acid, and tomentin from *J. podagrica.* This research provided practical information about purification of fraxetin by effective and simple methods. However, further work is required to establish more efficacious solvent systems to purify the methyl gallate, tomentin, and gallic acid in the *J. podagrica* stem bark.

## 4. Materials and Methods

### 4.1. Materials

*J. podagrica* Hook stem barks were collected from Thanh Hoa province, Vietnam (19° 48’ 24.0912’’ N and 105° 47’ 6.6552’’ E) in August 2017. The identification of the plant was authenticated by the corresponding author [42]. The samples were preliminarily sterilized by NaOCl 1% and washed several times with water. After drying in an oven at 50 °C for 10 days, a sample with voucher number ND-JP-VNB1 was preserved at the Laboratory of Plant Physiology and Biochemistry, Hiroshima University.

### 4.2. Extraction of Jatropha podagrica Stem Bark

The powder (2.3 kg) was immersed in 8 L of methanol (MeOH) for two weeks at room temperature. After filtration, the filtrate was concentrated under vacuum at 45 °C using a rotary evaporator (SB-350-EYELA, Tokyo Rikakikai Co., Ltd., Tokyo, Japan) to produce 50.8 g of crude extract. The crude extract was then suspended in distilled water (300 mL) and successively fractionated with hexane and ethyl acetate (EtOAc). The obtained amount of water, hexane, and EtOAc extracts were 22.6, 15.2, and 13 g, respectively. After screening the biological activities of extracts (Table 1), the EtOAc extract was used for isolation of the bioactive compounds using column chromatography (Figure 1).

### 4.3. Compounds Isolation from Ethyl Acetate Extract

The ethyl acetate extract was subjected to normal phase column chromatography (CC) over silica gel (200 g) of 70–230 mesh ASTM (Merck, Darmstadt, Germany) and LiChroprep RP-18 (Merck KGaA, Darmstadt, Germany) (40–63 mm). All fractions were examined by thin-layer chromatography (TLC) (Merck, Darmstadt, Germany). In TLC analysis, TLC Silica gel 60 was used as a solid phase and a mixture of the solvents hexane: ethyl acetate 8:2 (v:v) was the mobile phase. Fractions 20–38 (**C1**) (233 mg), fractions 51–95 (**C2**) (200 mg), fractions 96–142 (**C3**) (282 mg), fractions 143–196 (**C4**) (198 mg), fractions 197–204 and filtered 143–196 (**C5**) (30 mg), were crystallized after separation by CC (Figure 1). Crystal compound **C5** was obtained as a pure compound, the purity levels were confirmed by GC-MS of 97.67%.

### 4.4. DPPH Radical Scavenging Assay

The free radical scavenging activity was performed following the method by Elzaawely et al. [43]. A volume of 0.5 mL extract was mixed with 0.25 mL of 2,2-diphenyl-1-picrylhydrazyl (DPPH) solution and 0.1 mL of 0.1 M acetate buffer pH (5.5). The mixture was shaken and kept at room temperature for 30 min in the dark. The absorbance was measured at 517 nm using a microplate reader (MultiskanTM Microplate Spectrophotometer, Thermo Fisher Scientific, Osaka, Japan). The BHT standard (5–20 ppm) was used as the positive reference. The inhibition concentration (IC_50_) was the concentration of the samples which gave 50% DPPH radical scavenging activity. Thus, a lower IC_50_ value indicated a higher antioxidant activity. The following formula measured the percentage of DPPH radical scavenging activity.

% radical scavenging activity = [(A_control_ − A_test_)/A_control_] × 100

A_control_ corresponds to the absorbance of the control and A_test_ corresponds to the absorbance of the test extract. The IC_50_ value was also calculated using percent radical scavenging activity. Lower IC_50_ values indicate higher antioxidant activity.

### 4.5. Radical Cation ABTS Decolorization Assay

The ABTS (2,2′-azinobis-(3-ethylbenzothiazoline-6-sulfonic acid)) radical cation decolorization assay was carried out as an improved ABTS method of Re et al. [44], with some adjustments. Briefly, the ABTS radical solution was prepared by mixing 7 mM ABTS (2,20-azinobis (3-ethylbenzothiazoline-6-sulfonic acid)) and 2.45 mM potassium persulfate in water. The solution was incubated in the dark at room temperature for 16 h, and then diluted with methanol to obtain an absorbance of 0.70 ± 0.05 at 734 nm. An aliquot of 120 μL of the ABTS solution was mixed with 24 μL of a sample and the mixture was incubated in the dark at room temperature for 30 min. The absorbance of the reaction was recorded at 734 nm using a spectrophotometer (MultiskanTM Microplate Spectrophotometer, Thermo Fisher Scientific, Osaka, Japan). BHT standard solutions (0.01–0.25 mg/mL) were prepared and used as a positive control. The percentage inhibition was calculated according to the formula:

ABTS radical scavenging activity (%) = [(Abs_control_ − Abs_sample_)/A_control_] × 100

The Abs_control_ is the absorbance of the ABTS radical solution without samples and the Abs_sample_ is the absorbance of ABTS radical solution with samples.

### 4.6. Ferric Reducing Antioxidant Power Test (FRAP)

The reducing power was measured by the method as reported in Tuyen et al. [45]. Briefly, an aliquot of 0.1 mL of the extract was mixed with 2.5 mL potassium ferricyanide (1%) and 2.5 mL of phosphate buffer (0.2 M, pH 6.5). After incubation at 50 °C for 30 min, 2.5 mL of trichloroacetic acid (10%) was added to the mixture. The mixture was centrifuged at 4000 rpm for 10 min, and an aliquot of 2.5 mL of the supernatant was subsequently taken and mixed with 2.5 mL of distilled water and 0.5 mL FeCl_3_ (0.1%). The absorbance was measured at 700 nm using a microplate reader (Multiskan^TM^ Microplate Spectrophotometer, Thermo Fisher Scientific, Osaka, Japan). The BHT standard (5–20 ppm) was used as the positive control. The IC_50_ value was calculated.

### 4.7. Germination and Growth Bioassay

Growth suppressing potential of isolated fractions was assayed for radish (*R. sativus*), barnyard grass (*E. crus-galli*), lettuce (*L. sativa*.) seeds in an incubator (Biotron NC system, Nippon Medical & Chemical Instrument, Co. Ltd., Osaka, Japan). Photoperiodic was set up at day/night 12/12 h with temperature 25/23 °C. Each sample was diluted in methanol (MeOH) to obtain different concentrations (10, 100, 500, 1000, and 2000 µg/mL). The test solution (100 µL) was applied on filter papers lined in 96 well-plates and then ten seeds per well were tested (each well has 22 mm diameter × 18 mm height). After MeOH evaporation at room temperature, healthy seed was placed in a well, followed by the addition of 100 µL of distilled water. Plant germination monitoring was performed every 24 h for seven days. This bioassay was replicated six times (n = 6). The growth parameters of radicle (root) and hypocotyl (shoot) length were measured. Concentration in reducing 50% shoot and root lengths (IC_50_) was also calculated according to Xuan et al. [46].

### 4.8. Determination of Minimum Inhibitory Concentration (MIC)

Antibacterial activity of *J. podagrica* stem bark was measured using the disk diffusion method described by Ribeiro et al. [47] with minor modifications. The microorganisms used for this experiment were *K. pneumoniae*, *S. aureus*, *L. monocytogenes*, *B. subtilis*, *E. coli*, and *P. mirabilis*. Active cultures were prepared by transferring microbial inoculum from stock cultures to a test tube containing Muller–Hinton Broth, followed by incubation at 37 °C for 24 h. The bacterial inoculum was adjusted to achieve a turbidity equivalent to a 0.5 McFarland turbidity standard (10^6–8^ CFU/mL) by using sterile 0.85% saline. A volume of 0.1 mL of bacteria suspension was covered evenly on each Muller–Hinton agar dish. Next, samples were diluted in MeOH to obtain different concentrations ranging from 1.25 to 40 mg/mL (40, 30, 25, 20, 10, 5, 2.5, 1.5, 1.25 mg/mL). After that, filter paper dishes (6 mm diameter) impregnated with 20 µL of each sample were placed on the surface of agar plates. The incubation was maintained at 37 °C for 18–24 h in an ambient air incubator. The lowest concentration that inhibited the visible bacterial growth was determined as the minimum inhibitory concentration (MIC). Streptomycin and ampicillin (1.25, 0.625, 0.313, 0.156, 0.078, 0.039, 0.0195, 0.0097, 0.0048, 0.0024, 0.0012, 0.0006 mg/mL) were used as positive control in this experiment. MeOH was used as a negative control [48,49].

### 4.9. Chemical Constituents Identification by Gas Chromatography-Mass Spectrometry (GC-MS)

GC-MS analysis was performed to determine the chemical constituents of **C1**, **C2**, **C3**, **C4**, and **C5.** The GC-MS system was equipped with a DB-5MS column (30 m × 0.25 mm internal diameter × 0.25 µm in thickness (Agilent Technologies, J & W Scientific Products, Folsom, CA, USA). The carrier gas was helium and the split ratio was 5:1. The GC oven temperatures operated with the initial temperature of 50 °C without hold time, followed by an increase of 10 °C/min up to a final temperature of 300 °C and holding time 20 min. The carrier gas was helium at a flow rate of 1 mL/min. The injector and detector temperature were programmed at 300 °C and 320 °C, respectively. The mass was scanned from 29 to 800 amu. JEOL’s GC-MS Mass Center System Version 2.65a was used to control the GC-MS system and process the data peak [50]. Identification of volatiles was performed comparing their mass spectra with those of NIST/EPA/NIH Mass Spectral Library 2014 coupled with the GC-MS system. Wiley-WHC, Weinheim, Germany [23], which included the Retention Index Library, had 82,868 compounds. In addition, a standard solution of C7–C40-alkanes was used to obtain the retention index of compounds.

Quantification of the pure compound (**C5**) was done using the method described previously [51]. Fraxetin was dissolved in MeOH to obtain various concentrations of 5, 10, 50 ppm. The operation of GC analysis was the same as the method described above. The retention time and areas of the standards and samples were compared to achieve standard curves (1.0 > r^2^ > 0.9). The content value of quantified compounds was expressed in milligrams per gram of dry weight (mg/g DW).

### 4.10. Electrospray Ionization-Mass Spectrometry (ESI-MS) and Liquid Chromatography-Electrospray Ionisation Mass Spectrometry (LC-ESI-MS) Analyses

ESI-MS analysis was conducted on negative/positive ion mode. Mass spectral characterization was performed using a LTQ Orbitrap XL mass spectrometer (Thermo Fisher Scientific, San Jose, CA, USA) connected with an electrospray ionization (ESI) source in negative (between *m*/*z* 120 and 2000) and positive (between *m*/*z* 100 and 2000) ionization mode recording spectra. The instrumental conditions were as follows: spray voltage, 5.0 kV; sheath gas flow, 50 arb (arbitrary unit); aux gas flow rate, 10 arb; capillary temperature, 330 °C; capillary voltage, 50 V; tube lens, 80V [52].

LC-ESI-MS analysis was also implemented by using a LTQ Orbitrap XL (Thermo Fisher Scientific, San Jose, CA, USA) with a J-Pak Symphonia C18 (5 μm, 250 mm × 4.6 mm i.d.) column (JASCO Engineering Co., LTD, Tokyo, Japan). Mobile phase comprised (A) 0.1% formic acid in water (*v*/*v*) and (B) 0.1% formic acid in acetonitrile (*v*/*v*). Isocratic elution was accomplished with a mixture of A 30% and B 70%. The flow rate was adjusted to 0.4 mL/min within 30 min. The detector was set at 210 nm. The injection volume was 5 µL. Data acquisition was executed on ChromNAV software (JASCO, Tokyo, Japan). ESI conditions were: ion spray voltage, 4.5 kV; sheath gas flow rate, 60; aux gas flow rate, 20; capillary temperature, 350 °C; capillary voltage, 50 V; tube lens, 80V. MS analyses were run by a positive (*m*/*z* 100–1000) Fourier transform mass spectrometer (FTMS) at a resolution of 60000 and negative (*m*/*z* 115–1000) Ion trap mobility spectrometry (ITMS).

### 4.11. Nuclear Magnetic Resonance (NMR) Data of Fraxetin

*Fraxetin:*^1^H-NMR (MeOD, 600 MHz) *δ*: 6.20 (1H, d, *J* = 9.6 Hz, H-3), 7.83 (1H, d, *J* = 9.6 Hz, H-4), 6.73 (1H, s, H-5), 3.88 (3H, s, OCH_3_, H-6). ^13^C-NMR (MeOD, 150 MHz) *δ*: 163.71 (C-2), 112.68 (C-3), 146.73 (C-4), 101.07 (C-5), 140.65 (C-6), 147.12 (C-7), 140.73 (C-8), 134.07 (C-9), 112.16 (C-10), 56.79 (C-6-OCH_3_) (Supplementary Material Figure S22 and S23), and compared with literature [53].

### 4.12. Statistical Analysis

All obtained data were analyzed by Minitab Software (version 16.0, copyright 2015, Minitab Inc., State College, PA, USA). Two-way analysis of variance (ANOVA) and Tukey’s post hoc test were used to identify the significant difference among mean values with *p* < 0.05. All trials were designed randomly in triplicate.

## 5. Conclusions

In this study, the combined dilution of hexane and ethyl acetate at ratios 8:2, 7:3, and 6:4 by column chromatography successfully purified active compounds in stem bark of *J. podagrica*. This was the first time of successfully isolating and identifying gallic acid, methyl gallate, fraxetin, and tomentin from the medicinal plant. Results of in vitro assays showed that these constituents possessed potential antioxidant, antibacterial, and plant growth inhibitory properties.

## Figures and Tables

**Table 1:** DPPH, ABTS, and reducing power (FRAP) of solvent extractions from the stem bark of *Jatropha podagrica* in terms of IC_50_ values.

Name	DPPH IC_50_ (µg/mL)	ABTS IC_50_ (µg/mL)	Reducing Power IC_50_ (µg/mL)
BHT	9.3 ± 1.1 c	45.8 ± 1.5 b	426.8 ± 0.8 c
Hexane	262.1 ± 11.1 a	68.2 ± 4.9 b	555.4 ± 2.4 a
EtOAc	46.7 ± 2.0 b	66.0 ± 1.4 b	492.6 ± 6.2 ab
Aqueous	224.9 ± 16.1 a	144.2 ± 18.0 a	614.9 ± 0.4 a

Values are means ± SD (standard deviation). Values with similar letters in a column are not significantly different; (*p* < 0.05) according to Tukey’s post hoc test.

**Table 2:** Bioactive compounds identified in EtOAc extract of *Jatropha podagrica* stem bark by GC-MS.

Fraction	Retention Time	Peak Area (%)	Compounds	Chemical Formula	Molecular Weight	Chemical Class	Retention Index *
**C1**	10.35	4.76	Gallic acid	C_7_H_6_O_5_	170	Phenolic acid	1754
	16.37	96.24	Methyl gallate	C_8_H_8_O_5_	184	Phenol	1722
**C2**	10.35	12.09	Gallic acid	C_7_H_6_O_5_	170	Phenolic acid	1754
	16.37	3.87	Methyl gallate	C_8_H_8_O_5_	184	Phenol	1722
	18.95	84.04	Fraxetin	C_10_H_8_O_5_	208	Coumarin	2004
**C3**	16.37	2.94	Methyl gallate	C_8_H_8_O_5_	184	Phenol	1722
	18.35	54.34	Tomentin	C_11_H_10_O_5_	222	Coumarin	2085
	18.92	42.72	Fraxetin	C_10_H_8_O_5_	208	Coumarin	2004
**C4**	16.35	12.32	Methyl gallate	C_8_H_8_O_5_	184	Phenol	1722
	18.96	87.68	Fraxetin	C_10_H_8_O_5_	208	Coumarin	2004
**C5**	18.96	97.67	Fraxetin	C_10_H_8_O_5_	208	Coumarin	2004

* The retention indices were obtained from the NIST/EPA/NIH Mass Spectral Library 2014. Wiley-WHC, Weinheim, Germany [23].

**Table 3:** Quantity of fraxetin from *Jatropha podagrica* stem bark.

Fraction	Retention time	Compounds	Concentration (µg/g DW)
**C5**	18.96 ± 0.02	Fraxetin	13.04 ± 0.13

DW: dry weight. Values are means ± SD (standard deviation) (n = 3).

**Table 4:** Antioxidant activity measured by DPPH, ABTS, and reducing power of EtOAc extract fractions from stem bark of *Jatropha podagrica* in term of IC_50_ values.

Fraction	DPPH IC_50_ (µg/mL)	ABTS IC_50_ (µg/mL)	Reducing power IC_50_ (µg/mL)
BHT	9.30 ± 1.13 b	45.77 ± 1.45 a	426.73 ± 0.81 a
**C1**	40.83 ± 4.16 a	21.83 ± 2.21 b	391.77 ± 6.46 c
**C2**	2.50 ± 2.06 c	2.50 ± 0.20 d	387.57 ± 4.42 cd
**C3**	6.92 ± 6.64 b	7.52 ± 0.80 c	401.80 ± 1.71 b
**C4**	3.54 ± 3.07 c	3.36 ± 0.30 d	384.27 ± 1.12 cd
**C5**	2.81 ± 2.17 c	3.23 ± 0.30 d	381.10 ± 1.57 d

Values are means ± SD (standard deviation). Values with similar letters in a column are not significantly different; (*p* < 0.05) according to Tukey’s post hoc test.

**Table 5:** Antibacterial activity in term of minimum inhibitory concentration (MIC)values of fractions from stem bark of *Jatropha podagrica.*

Fractions	Minimum Inhibitory Concentration (mg/mL)					
	*S. aureus*	*E. coli*	*K. pneumoniae*	*L. monocytogenes*	*B. subtilis*	*P. mirabilis*
**C1**	10	25	nd	25	40	25
**C2**	10	25	nd	25	30	25
**C3**	10	20	30	20	40	20
**C4**	5	20	30	20	25	20
**C5**	10	20	25	25	30	25
MeOH *	-	-	-	-	-	-
Ampicillin **	0.0012	0.0098	0.0195	0.0049	0.0195	0.039
Streptomycin **	0.156	0.156	0.156	0.078	0.156	0.156

-: no inhibition, *: negative control, **: positive control.

**Table 6:** Inhibitory effects of isolated compounds from stem bark of *Jatropha podagrica* on growth of *R. sativus*, *E. crus-galli*, and *L. sativa.*

Fraction	*R. sativus* IC_50_ (µg/mL)		*E. crus-galli* IC_50_ (µg/mL)		*L. sativa* IC_50_ (µg/mL)	
	Shoot	Root	Shoot	Root	Shoot	Root
**C1**	1272.0 ± 32.7 b	194.0 ± 4.4 d	495.7 ± 5.1 d	219.7 ± 8.8 b	195.5 ± 5.5 c	57.9 ± 1.0 c
**C2**	924.7 ± 48.5 c	212.3 ± 5.1 c	825.3 ± 5.0 c	264.7 ± 47.4 b	499.5 ± 10.0 b	389.9 ± 4.0 b
**C3**	1615.8 ± 161.5 a	409.3 ± 9.1 b	1629.2 ± 10.1 a	143.9 ± 5.4 c	49.4 ± 3.5 d	47.1 ± 2.0 d
**C4**	1231.4 ± 216.8 bc	402.5 ± 6.7 b	854.8 ± 4.7 b	391.8 ± 5.4 a	576.7 ± 6.0 a	626.8 ± 5.0 a
**C5**	1201.8 ± 7.0 bc	436.1 ± 6.0 a	846.2 ± 5.4 b	409.5 ± 6.8 a	485.3 ± 5.0 b	30.0 ± 1.0 e

Values are means ± SD (standard deviation). Values with similar letters in a column are not significantly different; (*p* < 0.05) according to Tukey’s post hoc test.

**Table 7:** Antibacterial capacities of identified compounds from *Jatropha podagrica* by previous researches (concentration 20 µg/dish).

Compounds	Inhibition zone (mm)				Reference
	*B. subtilis*	*S. aureus*	*E. coli*	*P. aeruginosa*	
Fraxidin	12.0	-	-	-	[13]
Erythrinasinate	15.0	-	-	-	
Japodic acid	-	-	-	-	
Japodagrin	16.0	12.0	-	-	[7]
Japodagrone	12.0	-	-	-	
4z-Jatrogrosidentadion	20.0	10.0	-	-	
15-epi-4z–Jatrogrossidentadion	17.0	9.0	-	-	
2-Hydroxyisojatrogrossidion	31.0	21.0	-	-	
2-epi-Hydroxyisojatrogrossidion	35.0	26.0	-	-	

**-**: inactive.

## Supplementary Materials

Figures S1–S23 are available online.
