# Supplementary material for: Isolation and Purification of Bioactive Compounds from the Stem Bark of Jatropha podagrica
Source: Molecules. 2019 Mar 3;24(5):889. doi: 10.3390/molecules24050889 (PMC6429288; doi:10.3390/molecules24050889)
Supplement: Supplementary file 1 [file molecules-24-00889-s001.zip › Figure S22. 1H spectrum of fraction 5.pdf]

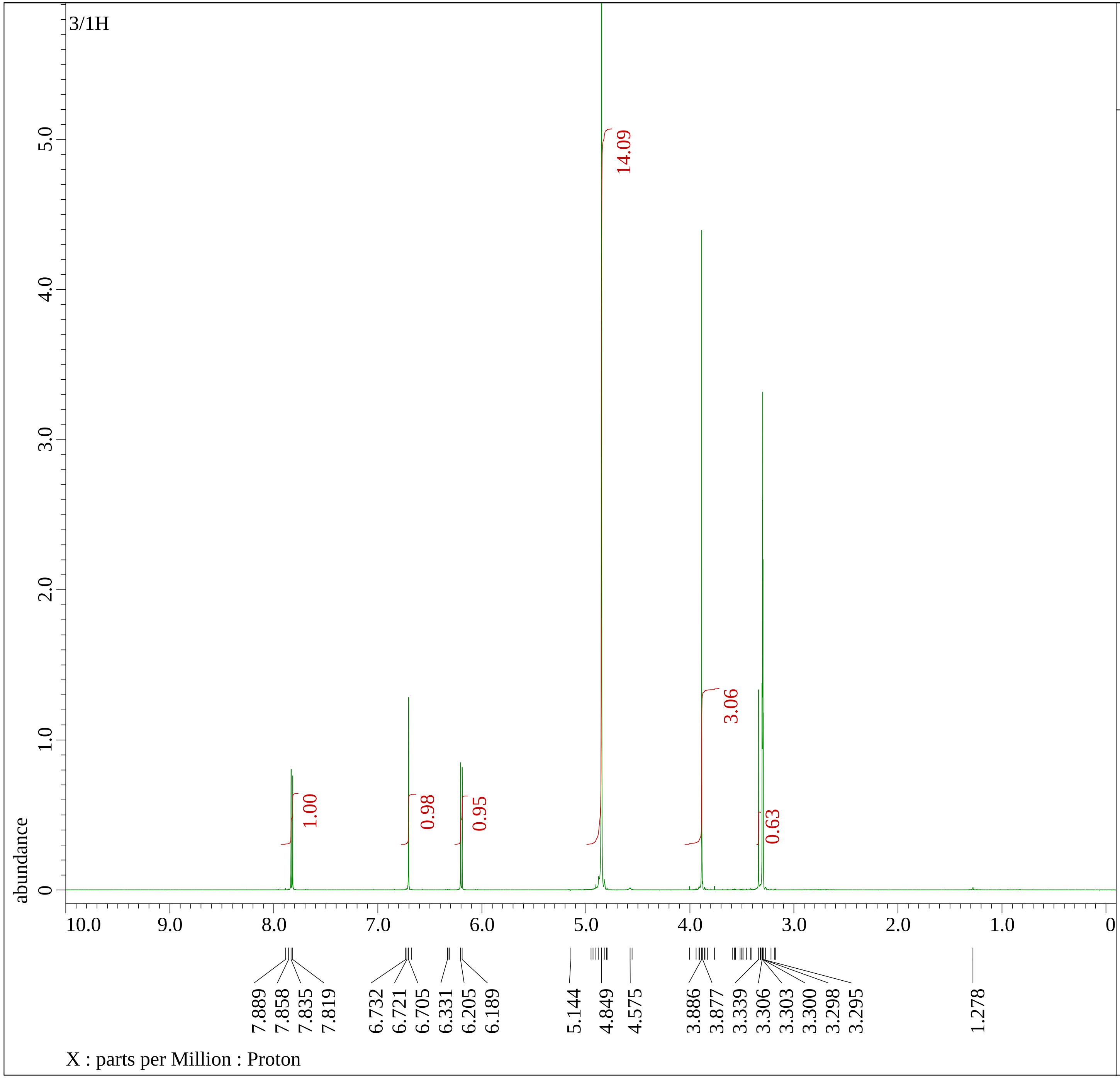

JEOL
Solutions for Innovation

```
Filename            = 171003_3_1H-1-4.jdf
Author              = delta
Experiment          = proton.jxp
Sample_Id           = 171003_3
Solvent             = METHANOL-D4
Creation_Time       =  4-OCT-2017 01:05:35
Revision_Time       =  4-OCT-2017 09:41:30
Current_Time        =  4-OCT-2017 09:42:03

Comment             = 3/1H
Data_Format         = 1D COMPLEX
Dim_Size            = 26214
Dim_Title           = Proton
Dim_Units           = [ppm]
Dimensions          = X
Spectrometer        = DELTA2_NMR

Field_Strength      = 14.09636928[T] (600[MHz]
X_Acq_Duration      = 2.9097984[s]
X_Domain            = 1H
X_Freq              = 600.1723046[MHz]
X_Offset            = 5[ppm]
X_Points            = 32768
X_Prescans          = 1
X_Resolution        = 0.34366642[Hz]
X_Sweep             = 11.26126126[kHz]
X_Sweep_Clipped     = 9.00900901[kHz]
Irr_Domain          = Proton
Irr_Freq            = 600.1723046[MHz]
Irr_Offset          = 5[ppm]
Tri_Domain          = Proton
Tri_Freq            = 600.1723046[MHz]
Tri_Offset          = 5[ppm]
Clipped             = FALSE
Scans               = 32
Total_Scans         = 32

Relaxation_Delay    = 5[s]
Recvr_Gain          = 50
Temp_Get            = 24[dC]
X_90_Width          = 13.5[us]
X_Acq_Time          = 2.9097984[s]
X_Angle             = 45[deg]
X_Atn               = 9.8[dB]
X_Pulse             = 6.75[us]
Irr_Mode            = Off
Tri_Mode            = Off
Dante_Presat        = FALSE
Initial_Wait        = 1[s]
Repetition_Time     = 7.9097984[s]
```
