# Supplementary material for: Isolation and Purification of Bioactive Compounds from the Stem Bark of Jatropha podagrica
Source: Molecules. 2019 Mar 3;24(5):889. doi: 10.3390/molecules24050889 (PMC6429288; doi:10.3390/molecules24050889)
Supplement: Supplementary file 1 [file molecules-24-00889-s001.zip › Figure S23. 13C spectrum of fraction 5.pdf]

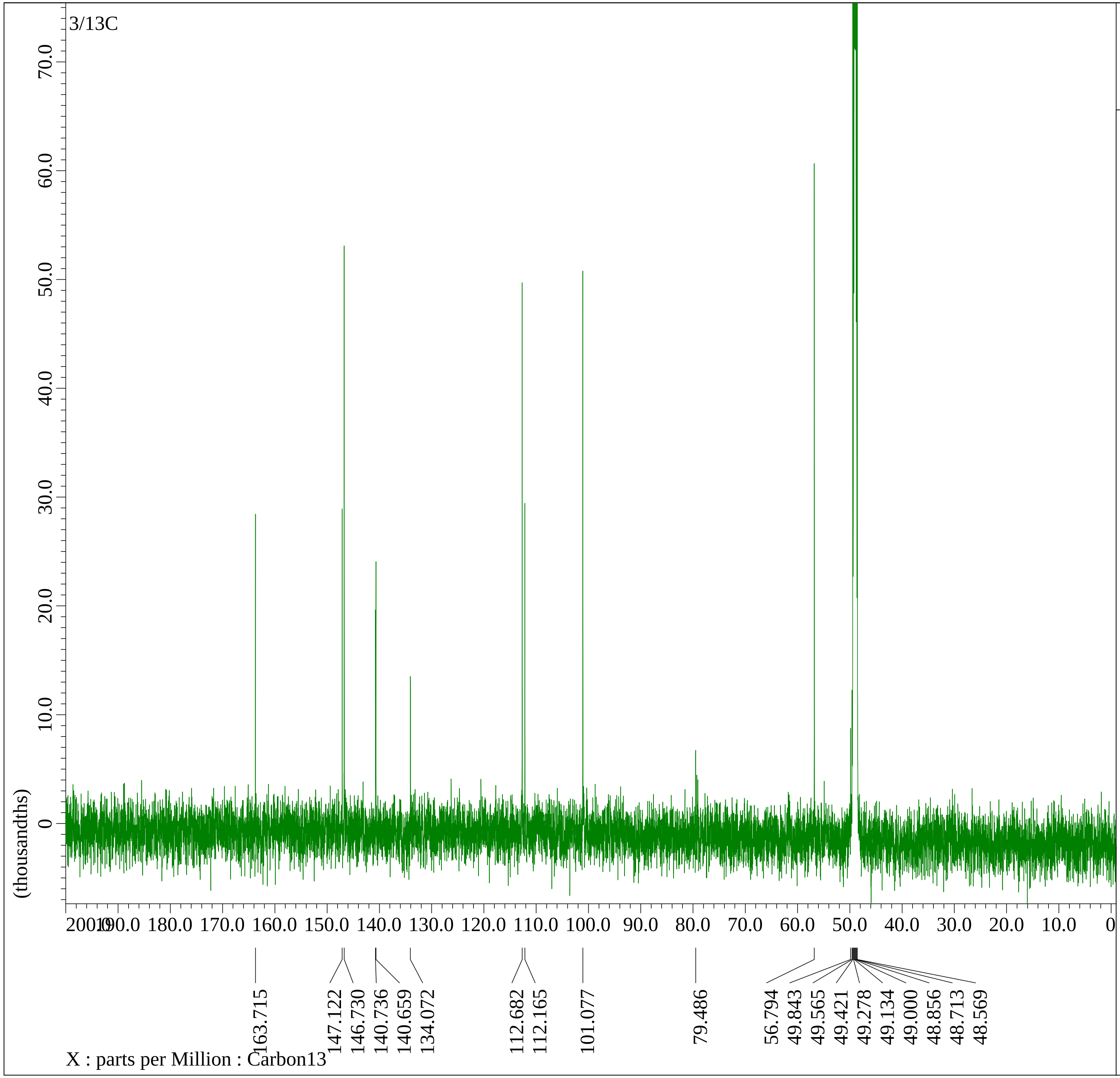


```
Filename          = 171003_3_13C-1-4.jdf
Author            = delta
Experiment        = carbon.jxp
Sample_Id         = 171003_3
Solvent           = METHANOL-D4
Creation_Time     =  4-OCT-2017 01:10:16
Revision_Time     =  4-OCT-2017 09:43:07
Current_Time      =  4-OCT-2017 09:43:43

Comment           = 3/13C
Data_Format       = 1D COMPLEX
Dim_Size          = 26214
Dim_Title         = Carbon13
Dim_Units         = [ppm]
Dimensions        = X
Spectrometer      = DELTA2_NMR

Field_Strength    = 14.09636928[T] (600[MHz]
X_Acq_Duration    = 0.69206016[s]
X_Domain          = 13C
X_Freq            = 150.91343039[MHz]
X_Offset          = 100[ppm]
X_Points          = 32768
X_Prescans        = 4
X_Resolution      = 1.44496109[Hz]
X_Sweep           = 47.34848485[kHz]
X_Sweep_Clipped   = 37.87878788[kHz]
Irr_Domain        = Proton
Irr_Freq          = 600.1723046[MHz]
Irr_Offset        = 5[ppm]
Clipped           = FALSE
Scans             = 4000
Total_Scans       = 4000

Relaxation_Delay  = 2[s]
Recvr_Gain        = 60
Temp_Get          = 24.7[dC]
X_90_Width        = 10.2[us]
X_Acq_Time        = 0.69206016[s]
X_Angle           = 30[deg]
X_Atn             = 11.1[dB]
X_Pulse           = 3.4[us]
Irr_Atn_Dec       = 24.81[dB]
Irr_Atn_Noe       = 24.81[dB]
Irr_Noise         = WALTZ
Irr_Pwidth        = 76[us]
Decoupling        = TRUE
Initial_Wait      = 1[s]
Noe               = TRUE
Noe_Time          = 2[s]
Repetition_Time   = 2.69206016[s]
```
