# Supplementary material for: Isolation and Purification of Bioactive Compounds from the Stem Bark of Jatropha podagrica
Source: Molecules. 2019 Mar 3;24(5):889. doi: 10.3390/molecules24050889 (PMC6429288; doi:10.3390/molecules24050889)

ESI-MS spectrum (Negative ion mode)

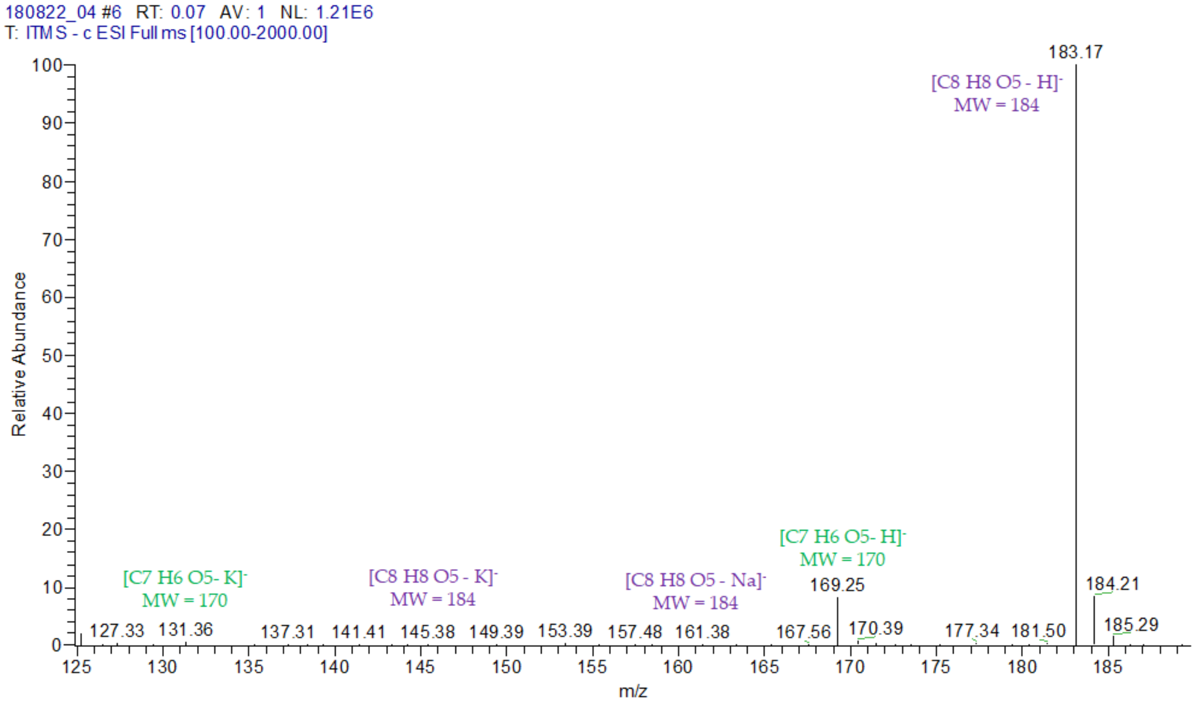

ESI-MS spectrum (Positive ion mode)

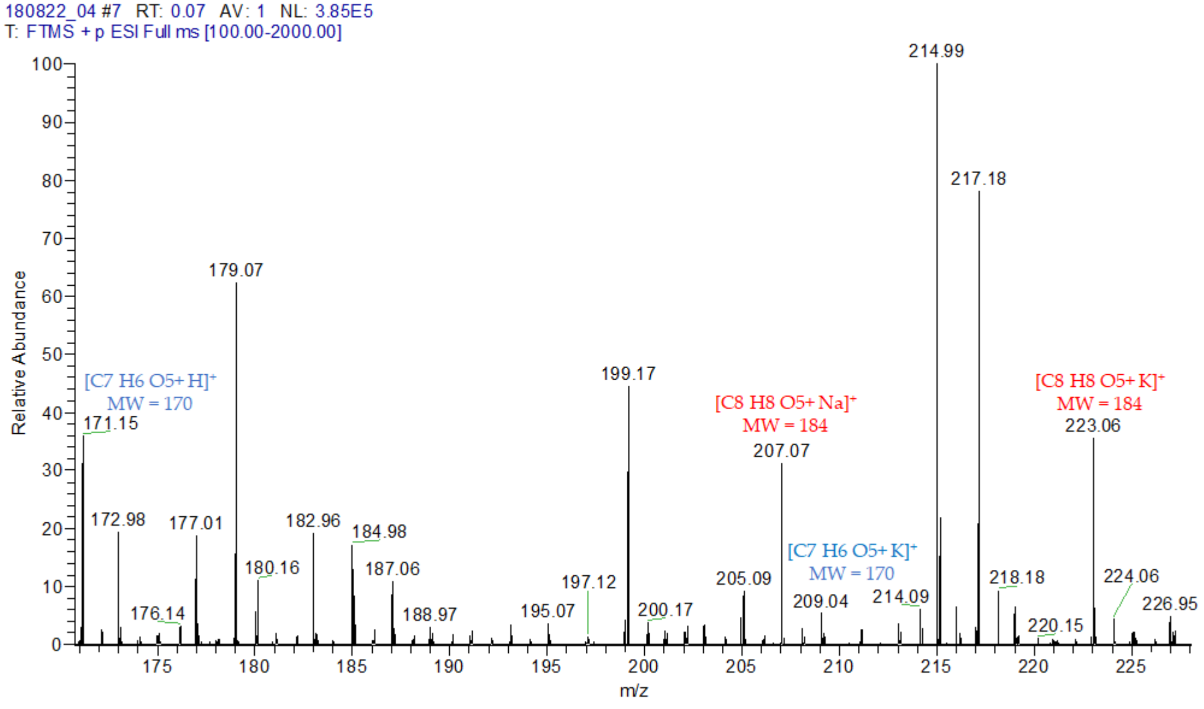

Supplement: Supplementary file 1 [file molecules-24-00889-s001.zip › Figure S3. ESI-MS spectrum of fraction 1.pdf]
