# Supplementary material for: Isolation and Purification of Bioactive Compounds from the Stem Bark of Jatropha podagrica
Source: Molecules. 2019 Mar 3;24(5):889. doi: 10.3390/molecules24050889 (PMC6429288; doi:10.3390/molecules24050889)
Supplement: Supplementary file 1 [file molecules-24-00889-s001.zip › Table S1. The fragmentation patterns and intensity data of fraction 1.pdf]

(Gallic acid: similarity 91%)

Peak	m/z	Relative intensity	Intensity
1	29.05	10.69	1350.84
2	53.05	9.74	1230.21
3	55.03	14.50	1831.55
4	79.08	78.18	9875.72
5	124.01	26.53	3351.81
6	152.02	100.00	12632.34
7	152.04	11.01	1391.06
8	170.12	64.37	8131.58

(Methyl gallate: similarity 87%)

Peak	m/z	Relative intensity	Intensity
1	24.04	2.90	37238.62
2	25.03	8.56	109820.44
3	26.03	1.59	20343.47
4	41.04	0.85	10840.25
5	42.01	1.52	19481.46
6	42.05	1.35	17289.62
7	43.02	0.82	10508.90
8	43.06	1.53	19648.10
9	44.06	1.37	17589.37
10	45.04	1.44	18524.35
11	53.04	0.82	10482.71
12	79.04	14.36	184163.28
13	85.02	1.83	23527.57
14	88.06	2.78	35675.12
15	95.07	1.60	20518.24
16	97.04	1.62	20716.61
17	107.08	5.56	71355.45
18	124.02	0.94	12042.39
19	125.05	2.65	33948.91
20	125.09	2.12	27144.36
21	126.03	1.68	21481.89
22	153.05	100.00	1282224.74
23	153.08	0.78	10055.75
24	158.02	1.29	16575.11
25	184.08	47.45	608413.28
