# Supplementary material for: Isolation and Purification of Bioactive Compounds from the Stem Bark of Jatropha podagrica
Source: Molecules. 2019 Mar 3;24(5):889. doi: 10.3390/molecules24050889 (PMC6429288; doi:10.3390/molecules24050889)
Supplement: Supplementary file 1 [file molecules-24-00889-s001.zip › Table S2. The fragmentation patterns and intensity data of fraction 2.pdf]

(Gallic acid: similarity 90%)

Peak	m/z	Relative intensity	Intensity
1	29.05	13.07	1650.83
2	53.05	9.74	1230.21
3	55.03	12.23	1544.34
4	79.08	78.95	9972.76
5	124.01	26.53	3351.81
6	152.02	100.00	12632.34
7	152.04	11.01	1391.06
8	170.12	65.87	8321.52

(Methyl gallate: similarity 88%)

Peak	m/z	Relative intensity	Intensity
1	24.04	3.32	42563.74
2	25.03	9.35	119831.45
3	26.03	1.81	23252.59
4	41.04	0.97	12390.41
5	42.01	1.74	22267.31
6	42.05	1.54	19762.04
7	43.02	0.94	12011.67
8	43.06	1.75	22457.78
9	44.06	1.57	20104.65
10	45.04	1.65	21173.33
11	53.04	0.93	11981.74
12	79.04	14.38	184323.28
13	85.02	2.10	26892.01
14	88.06	3.18	40776.66
15	95.07	1.83	23452.35
16	**97.04**	1.85	23679.09
17	107.08	6.36	81559.28
18	124.02	1.07	13764.45
19	125.05	3.03	38803.60
20	125.09	2.42	31026.00
21	126.03	1.91	24553.80
22	153.05	100.00	1282224.74
23	184.08	47.04	603213.28

(Fraxetin: similarity 92%)

Peak	m/z	Relative intensity	Intensity	Peak	m/z	Relative intensity	Intensity
1	29.04	11.08	70713	40	93.08	4.54	29000
2	39.03	4.48	28572	41	94.08	3.05	19455
3	40.03	0.82	5237	42	95.09	0.37	2349
4	41.04	2.80	17865	43	96.1	7.70	49150
5	42.01	0.62	3974	44	97.07	0.99	6294
6	42.05	2.99	19076	45	97.11	2.06	13176
7	43.02	3.75	23927	46	105.08	0.81	5196
8	43.06	7.86	50165	47	107.09	2.43	15491
9	45.04	1.01	6444	48	108.1	1.98	12628
10	51.03	20.90	133461	49	109.05	29.68	189496
11	53.05	11.58	73912	50	109.11	5.91	37749
12	54.05	0.91	5841	51	110.12	4.30	27461
13	55.02	2.34	14921	52	111.09	0.64	4078
14	55.06	7.60	48553	53	111.12	0.66	4225
15	56.07	2.31	14721	54	115.08	0.83	5287
16	57.08	1.96	12501	55	119.09	0.58	3690
17	59.02	7.02	44800	56	121.11	1.98	12628
18	59.05	2.19	13971	57	122.12	1.55	9912
19	65.04	2.69	17200	58	123.09	0.68	4350
20	66.05	2.35	14999	59	123.13	2.36	15040
21	67.06	3.60	22990	60	124.13	2.01	12857
22	68.07	1.97	12587	61	133.11	0.66	4184
23	69.08	10.69	68235	62	135.09	0.73	4640
24	70.08	1.04	6667	63	135.13	1.43	9152
25	71.06	0.60	3856	64	136.1	0.94	5998
26	73.07	1.01	6462	65	136.13	1.10	7051
27	74.04	5.64	35977	66	137.05	28.39	181274
28	77.05	4.93	31453	67	137.14	1.01	6441
29	78.05	1.21	7706	68	138.15	1.04	6608
30	79.06	8.27	52777	69	149.11	0.87	5575
31	80.07	6.61	42182	70	149.14	0.77	4923
32	81.05	32.68	208647	71	150.11	2.21	14140
33	82.08	11.32	72242	72	163.12	0.97	6190
34	83.06	0.69	4406	73	165.05	24.30	155156
35	83.09	4.06	25917	74	180.08	20.74	132423
36	85.07	0.96	6128	75	192.02	0.71	4534
37	87.05	2.52	16071	76	193.06	40.01	255460
38	91.06	3.71	23662	77	208.08	100.00	638259
39	92.07	0.66	4226	78	209.09	11.51	73494
