# Supplementary material for: Isolation and Purification of Bioactive Compounds from the Stem Bark of Jatropha podagrica
Source: Molecules. 2019 Mar 3;24(5):889. doi: 10.3390/molecules24050889 (PMC6429288; doi:10.3390/molecules24050889)
Supplement: Supplementary file 1 [file molecules-24-00889-s001.zip › Table S3. The fragmentation patterns and intensity data of fraction 3.pdf]

(Methyl gallate: similarity 92%)

Peak	m/z	Relative intensity	Intensity	Peak	m/z	Relative intensity	Intensity
1	24.04	3.23	41446.58	13	85.02	2.04	26186.19
2	25.03	9.35	119831.45	14	88.06	3.10	39706.41
3	26.03	1.77	22642.28	15	95.07	1.78	22836.80
4	41.04	0.94	12065.20	16	**97.04**	1.80	23057.59
5	42.01	1.69	21682.86	17	107.08	6.19	79418.62
6	42.05	1.50	19243.35	18	124.02	1.05	13403.18
7	43.02	0.91	11696.41	19	125.05	2.95	37785.14
8	43.06	1.71	21868.34	20	125.09	2.36	30211.67
9	44.06	1.53	19576.97	21	126.03	1.86	23909.34
10	45.04	1.61	20617.60	22	153.05	100.00	1282224.74
11	53.04	0.91	11667.26	23	184.08	0.87	11192.05
12	79.04	16.00	205151.81				

(Tomentin: similarity 90%)

Peak	m/z	Relative intensity	Intensity	Peak	m/z	Relative intensity	Intensity
1	29.04	5.11	78347.52	19	65.04	0.92	14088.74
2	39.03	2.04	31278.73	20	66.05	1.09	16646.44
3	40.03	3.34	51100.55	21	67.06	1.01	15514.19
4	41.04	9.39	143826.97	22	68.07	0.91	13968.80
5	42.01	3.25	49810.56	23	69.08	1.03	15727.72
6	42.05	1.51	23170.24	24	93.08	0.48	7399.04
7	43.02	1.54	23554.38	25	94.08	0.93	14279.56
8	43.06	3.71	56793.17	26	95.09	0.47	7171.46
9	45.04	4.64	71051.48	27	96.1	1.30	19927.04
10	51.03	5.15	78915.05	28	97.07	0.97	14907.02
11	53.05	5.82	89207.72	29	132.13	0.56	8552.46
12	54.01	12.96	198482.07	30	133.99	20.79	318571.72
13	55.02	0.82	12559.85	31	151.14	3.06	46813.20
14	55.06	0.91	13883.63	32	194.05	100.00	1532043.00
15	56.07	1.07	16337.83	33	195.03	4.71	72191.61
16	57.08	0.91	13873.33	34	208.08	6.98	106962.73
17	59.02	0.90	13719.52	35	209.02	19.52	299032.23
18	59.05	1.01	15505.23	36	225.05	26.99	413509.08

(Fraxetin: similarity 86%)

Peak	m/z	Relative intensity	Intensity	Peak	m/z	Relative intensity	Intensity
1	29.04	11.08	71413	40	93.08	4.54	29861
2	39.03	4.48	28854	41	94.08	3.05	20032
3	40.03	0.82	5288	42	95.09	0.37	2418
4	41.04	2.80	18042	43	96.1	7.70	50609
5	42.01	0.62	4014	44	97.07	0.99	6481
6	42.05	2.99	19265	45	97.11	2.06	13567
7	43.02	3.75	24164	46	105.08	0.81	5350
8	43.06	7.86	50662	47	107.09	2.43	15951
9	45.04	1.01	6508	48	108.1	1.98	13003
10	51.03	20.90	134782	49	109.05	29.68	195122
11	53.05	11.58	74644	50	109.11	5.91	38870
12	54.05	0.91	5899	51	110.12	4.30	28277
13	55.02	2.34	15069	52	111.09	0.64	4199
14	55.06	7.60	49033	53	111.12	0.66	4351
15	56.07	2.31	14867	54	115.08	0.83	5444
16	57.08	1.96	12624	55	119.09	0.58	3800
17	59.02	7.02	45244	56	121.11	1.98	13003
18	59.05	2.19	14109	57	122.12	1.55	10206
19	65.04	2.69	17370	58	123.09	0.68	4479
20	66.05	2.35	15148	59	123.13	2.36	15486
21	67.06	3.60	23217	60	124.13	2.01	13239
22	68.07	1.97	12711	61	133.11	0.66	4308
23	69.08	10.69	68911	62	135.09	0.73	4778
24	70.08	1.04	6733	63	135.13	1.43	9423
25	71.06	0.60	3894	64	136.1	0.94	6176
26	73.07	1.01	6526	65	136.13	1.10	7260
27	74.04	5.64	36333	66	137.05	28.39	186657
28	77.05	4.93	31765	67	137.14	1.01	6632
29	78.05	1.21	7783	68	138.15	1.04	6804
30	79.06	8.27	53299	69	149.11	0.87	5740
31	80.07	6.61	42599	70	149.14	0.77	5070
32	81.05	32.68	210712	71	150.11	2.21	14560
33	82.08	11.32	72957	72	163.12	0.97	6374
34	83.06	0.69	4450	73	165.05	24.30	159762
35	83.09	4.06	26173	74	180.08	20.74	136354
36	85.07	0.96	6188	75	192.02	0.71	4669
37	87.05	2.52	16230	76	193.06	40.01	263044
38	91.06	3.71	23896	77	208.08	100.00	638259
39	92.07	0.66	4226	78	209.09	11.51	73568
