# Supplementary material for: Isolation and Purification of Bioactive Compounds from the Stem Bark of Jatropha podagrica
Source: Molecules. 2019 Mar 3;24(5):889. doi: 10.3390/molecules24050889 (PMC6429288; doi:10.3390/molecules24050889)
Supplement: Supplementary file 1 [file molecules-24-00889-s001.zip › Table S4. The fragmentation patterns and intensity data of fraction 4.pdf]

(Methyl gallate: similarity 87%)

Peak	m/z	Relative intensity	Intensity
1	24.04	3.31	42399.86
2	25.03	9.56	122587.57
3	26.03	1.81	23163.05
4	41.04	0.96	12342.70
5	42.01	1.73	22181.57
6	42.05	1.54	19685.94
7	43.02	0.93	11965.42
8	43.06	1.74	22371.31
9	44.06	1.56	20027.24
10	45.04	1.64	21091.81
11	53.04	0.93	11935.60
12	79.04	16.37	209870.30
13	85.02	2.09	26788.47
14	88.06	3.17	40619.66
15	95.07	1.82	23362.05
16	97.04	1.84	23587.91
17	107.08	6.34	81245.24
18	124.02	1.07	13711.45
19	125.05	3.01	38654.19
20	125.09	2.41	30906.54
21	126.03	1.91	24459.26
22	153.05	100.00	1282224.74
23	184.08	0.89	11449.47

(Fraxetin: similarity 93%)

Peak	m/z	Relative intensity	Intensity	Peak	m/z	Relative intensity	Intensity
1	29.04	11.08	70706	40	93.08	4.54	29861
2	39.03	4.48	28569	41	94.08	3.05	19455
3	40.03	0.82	5236	42	95.09	0.37	2349
4	41.04	2.80	17864	43	96.1	7.70	49150
5	42.01	0.62	3974	44	97.07	0.99	6294
6	42.05	2.99	19074	45	97.11	2.06	13176
7	43.02	3.75	23925	46	105.08	0.81	5196
8	43.06	7.86	50160	47	107.09	2.43	15491
9	45.04	1.01	6443	48	108.1	1.98	12628
10	51.03	20.90	133448	49	109.05	29.68	189496
11	53.05	11.58	73905	50	109.11	5.91	37749
12	54.05	0.91	5840	51	110.12	4.30	27461
13	55.02	2.34	14920	52	111.09	0.64	4078
14	55.06	7.60	48548	53	111.12	0.66	4225
15	56.07	2.31	14720	54	115.08	0.83	5287
16	57.08	1.96	12499	55	119.09	0.58	3690
17	59.02	7.02	44796	56	121.11	1.98	12628
18	59.05	2.19	13970	57	122.12	1.55	9912
19	65.04	2.69	17198	58	123.09	0.68	4350
20	66.05	2.35	14998	59	123.13	2.36	15040
21	67.06	3.60	22988	60	124.13	2.01	12857
22	68.07	1.97	12586	61	133.11	0.66	4184
23	69.08	10.69	68229	62	135.09	0.73	4640
24	70.08	1.04	6666	63	135.13	1.43	9152
25	71.06	0.60	3856	64	136.1	0.94	5998
26	73.07	1.01	6461	65	136.13	1.10	7051
27	74.04	5.64	35973	66	137.05	28.39	181274
28	77.05	4.93	31450	67	137.14	1.01	6441
29	78.05	1.21	7706	68	138.15	1.04	6608
30	79.06	8.27	52772	69	149.11	0.87	5575
31	80.07	6.61	42177	70	149.14	0.77	4923
32	81.05	32.68	208626	71	150.11	2.21	14140
33	82.08	11.32	72234	72	163.12	0.97	6190
34	83.06	0.69	4406	73	165.05	24.30	155156
35	83.09	4.06	25914	74	180.08	20.74	132423
36	85.07	0.96	6127	75	192.02	0.71	4534
37	87.05	2.52	16069	76	193.06	40.01	255460
38	91.06	3.71	23660	77	208.08	100.00	638259
39	92.07	0.66	4226	78	209.09	11.51	73494
