# Supplementary material for: Isolation and Purification of Bioactive Compounds from the Stem Bark of Jatropha podagrica
Source: Molecules. 2019 Mar 3;24(5):889. doi: 10.3390/molecules24050889 (PMC6429288; doi:10.3390/molecules24050889)
Supplement: Supplementary file 1 [file molecules-24-00889-s001.zip › Table S5. The fragmentation patterns and intensity data of fraction 5.pdf]

(Fraxetin: similarity 97%)

Peak	m/z	Relative intensity	Intensity	Peak	m/z	Relative intensity	Intensity
1	29.04	11.08	70692	40	93.08	4.54	28991
2	39.03	4.48	28563	41	94.08	3.05	19449
3	40.03	0.82	5235	42	95.09	0.37	2348
4	41.04	2.80	17860	43	96.1	7.70	49135
5	42.01	0.62	3973	44	97.07	0.99	6292
6	42.05	2.99	19070	45	97.11	2.06	13172
7	43.02	3.75	23920	46	105.08	0.81	5194
8	43.06	7.86	50150	47	107.09	2.43	15486
9	45.04	1.01	6442	48	108.1	1.98	12624
10	51.03	20.90	133421	49	109.05	29.68	189439
11	53.05	11.58	73890	50	109.11	5.91	37738
12	54.05	0.91	5839	51	110.12	4.30	27453
13	55.02	2.34	14917	52	111.09	0.64	4077
14	55.06	7.60	48538	53	111.12	0.66	4224
15	56.07	2.31	14717	54	115.08	0.83	5285
16	57.08	1.96	12497	55	119.09	0.58	3689
17	59.02	7.02	44787	56	121.11	1.98	12624
18	59.05	2.19	13967	57	122.12	1.55	9909
19	65.04	2.69	17195	58	123.09	0.68	4349
20	66.05	2.35	14995	59	123.13	2.36	15035
21	67.06	3.60	22983	60	124.13	2.01	12853
22	68.07	1.97	12583	61	133.11	0.66	4183
23	69.08	10.69	68215	62	135.09	0.73	4639
24	70.08	1.04	6665	63	135.13	1.43	9149
25	71.06	0.60	3855	64	136.1	0.94	5996
26	73.07	1.01	6460	65	136.13	1.10	7049
27	74.04	5.64	35966	66	137.05	28.39	181220
28	77.05	4.93	31444	67	137.14	1.01	6439
29	78.05	1.21	7704	68	138.15	1.04	6606
30	79.06	8.27	52761	69	149.11	0.87	5573
31	80.07	6.61	42169	70	149.14	0.77	4922
32	81.05	32.68	208584	71	150.11	2.21	14136
33	82.08	11.32	72220	72	163.12	0.97	6188
34	83.06	0.69	4405	73	165.05	24.30	155109
35	83.09	4.06	25909	74	180.08	20.74	132383
36	85.07	0.96	6126	75	192.02	0.71	4533
37	87.05	2.52	16066	76	193.06	40.01	255383
38	91.06	3.71	23655	77	208.08	100.00	638259
39	92.07	0.66	4225	78	209.09	11.51	73472
