# Supplementary figures and images for: Isolation and Purification of Bioactive Compounds from the Stem Bark of Jatropha podagrica
Source: Molecules. 2019 Mar 3;24(5):889. doi: 10.3390/molecules24050889 (PMC6429288; doi:10.3390/molecules24050889)

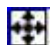

TIC: / EI+

MeOH

$\times 10^6$  Intensity (64965508)

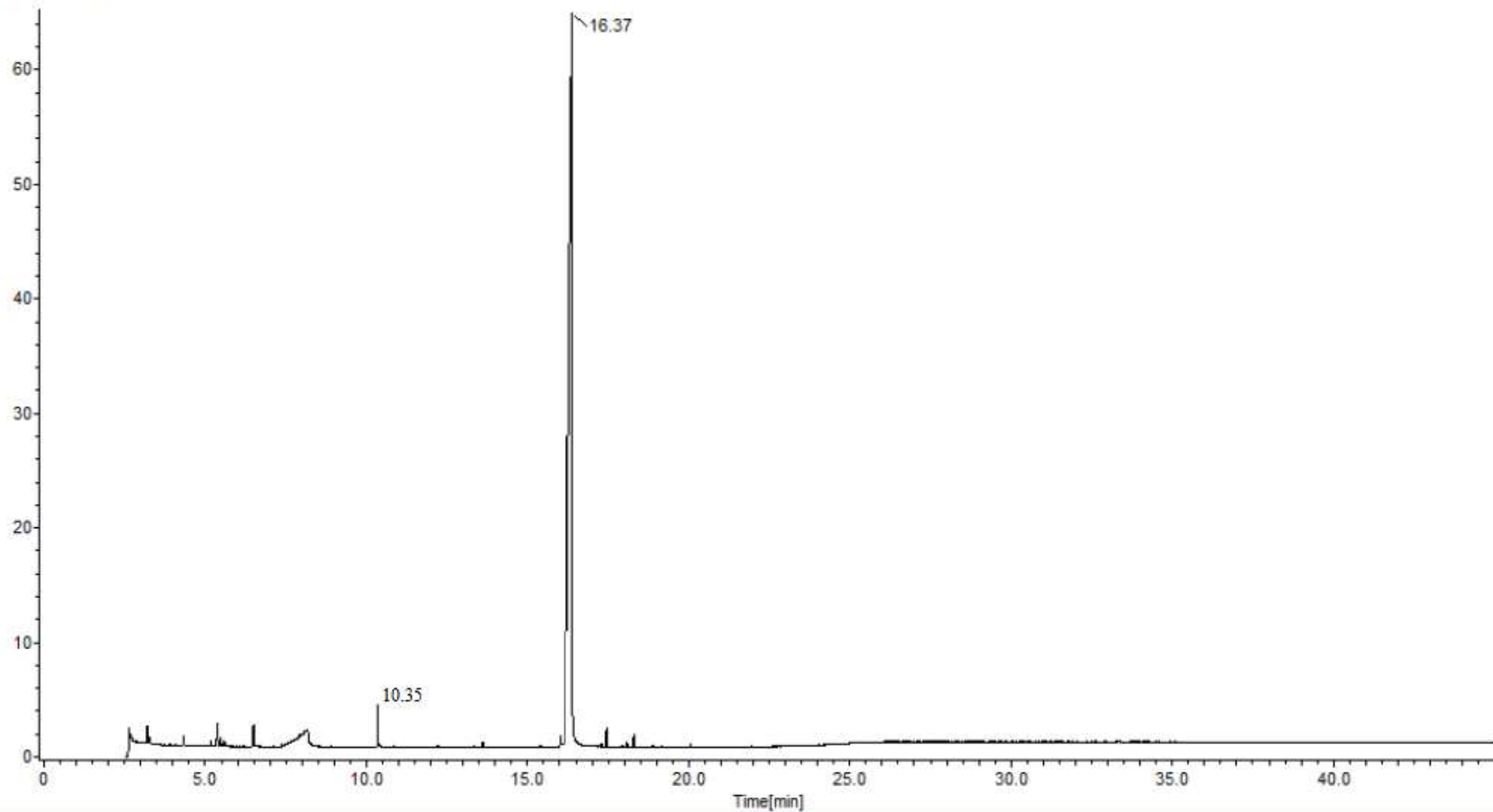

Supplement: Supplementary file 1 [file molecules-24-00889-s001.zip › Figure S1. GC-MS chromatogram of fraction 1.pdf]

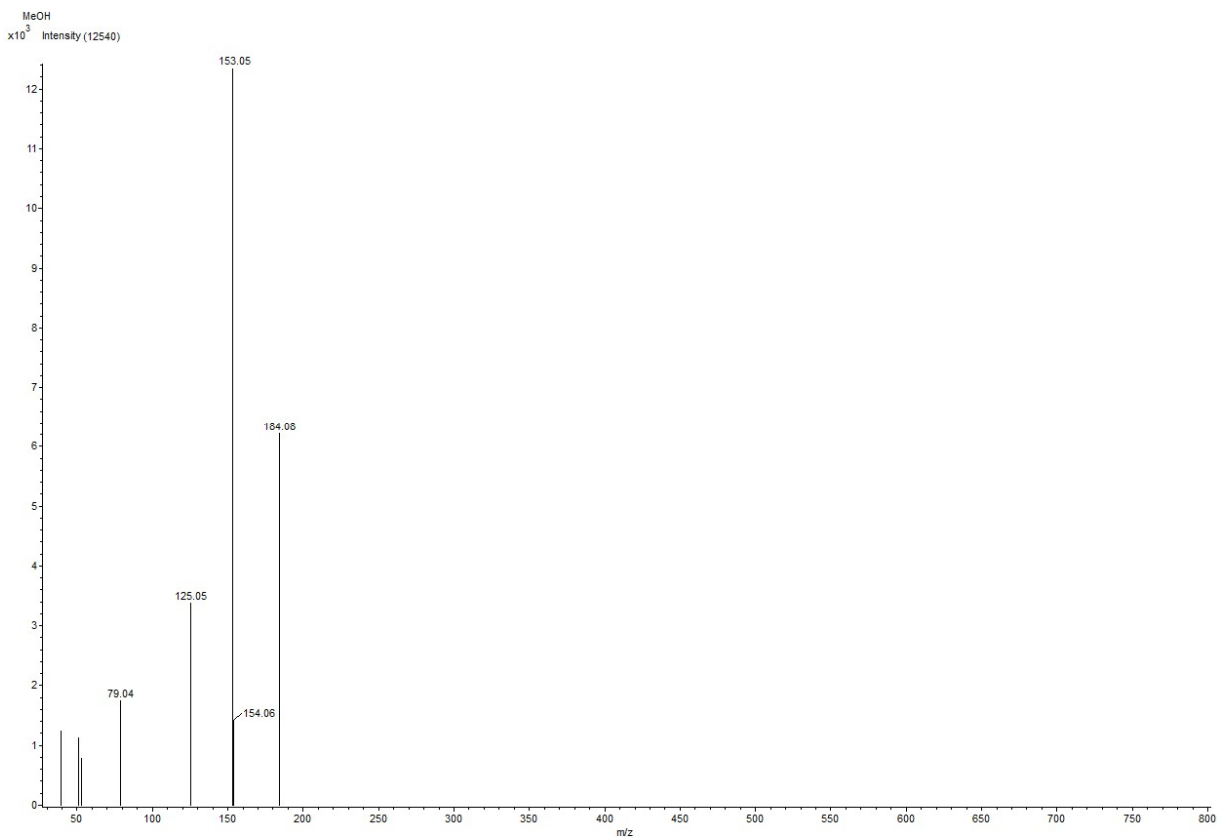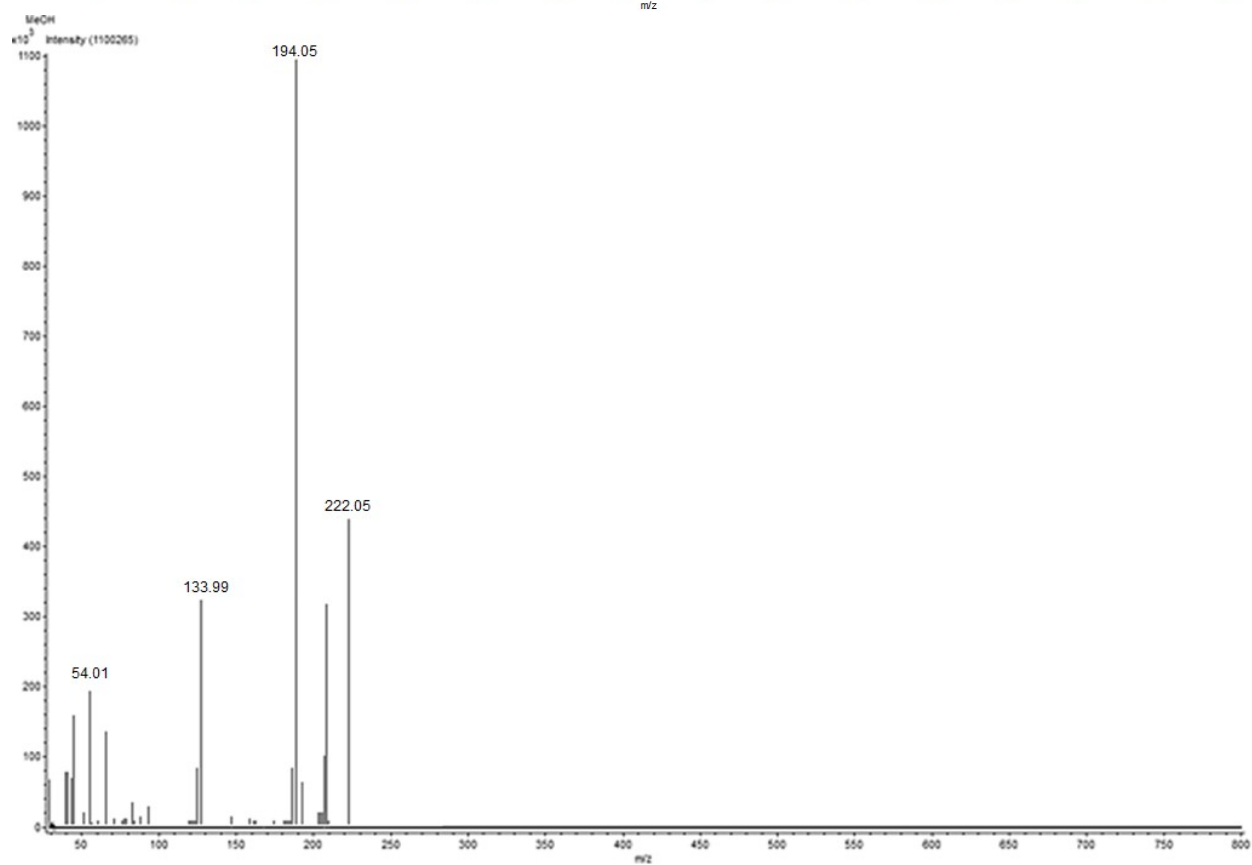

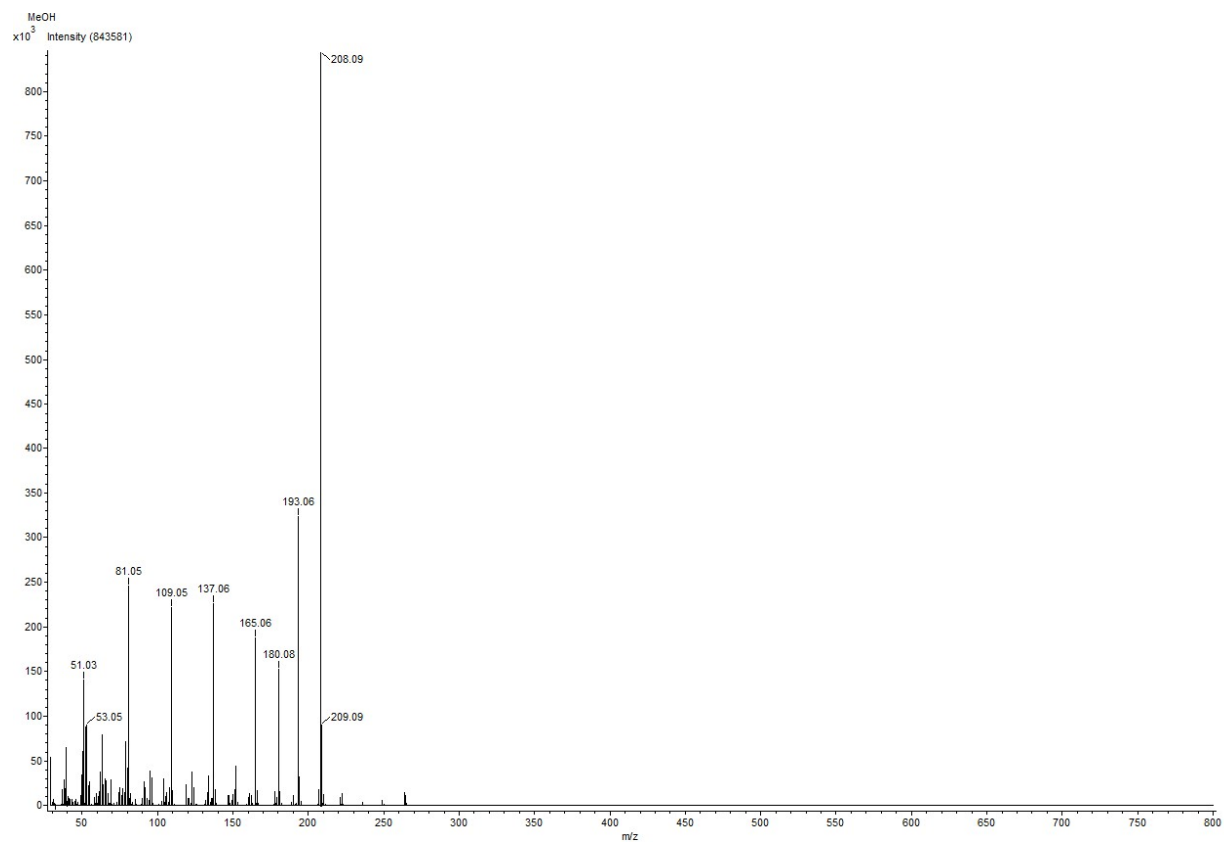

Spectrum c4

Supplement: Supplementary file 1 [file molecules-24-00889-s001.zip › Figure S10. GC-MS fragmentation of fraction 3.pdf]

## ESI-MS spectrum (Negative ion mode)

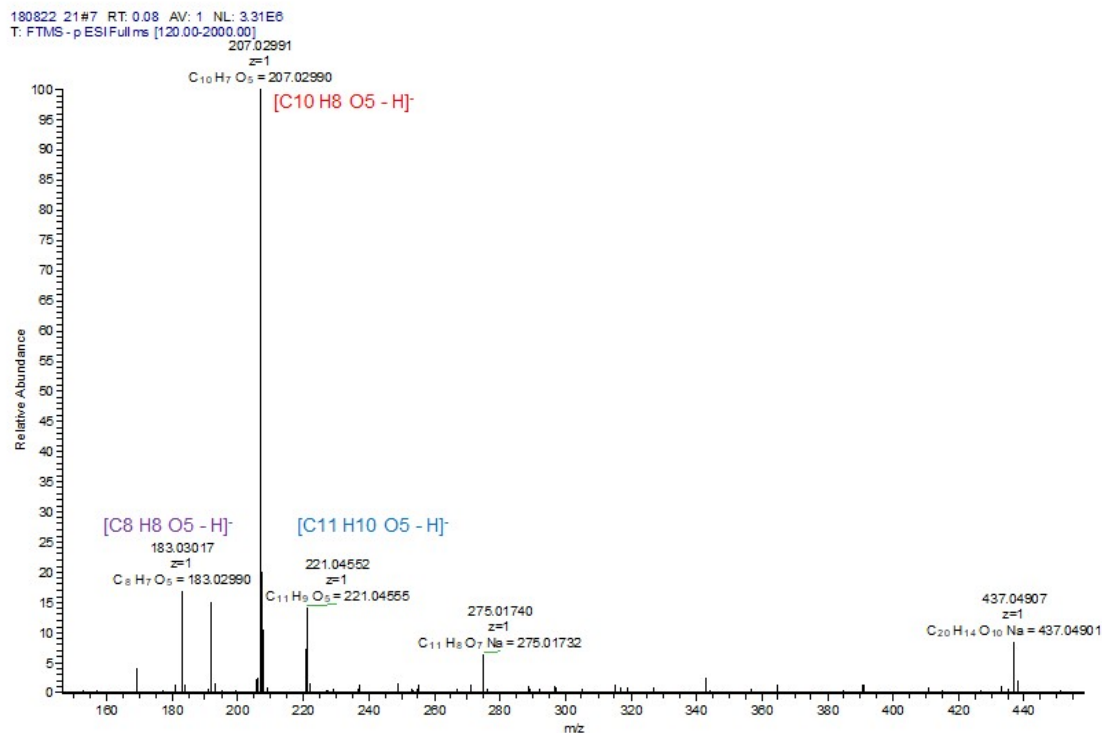

## ESI-MS spectrum (Positive ion mode)

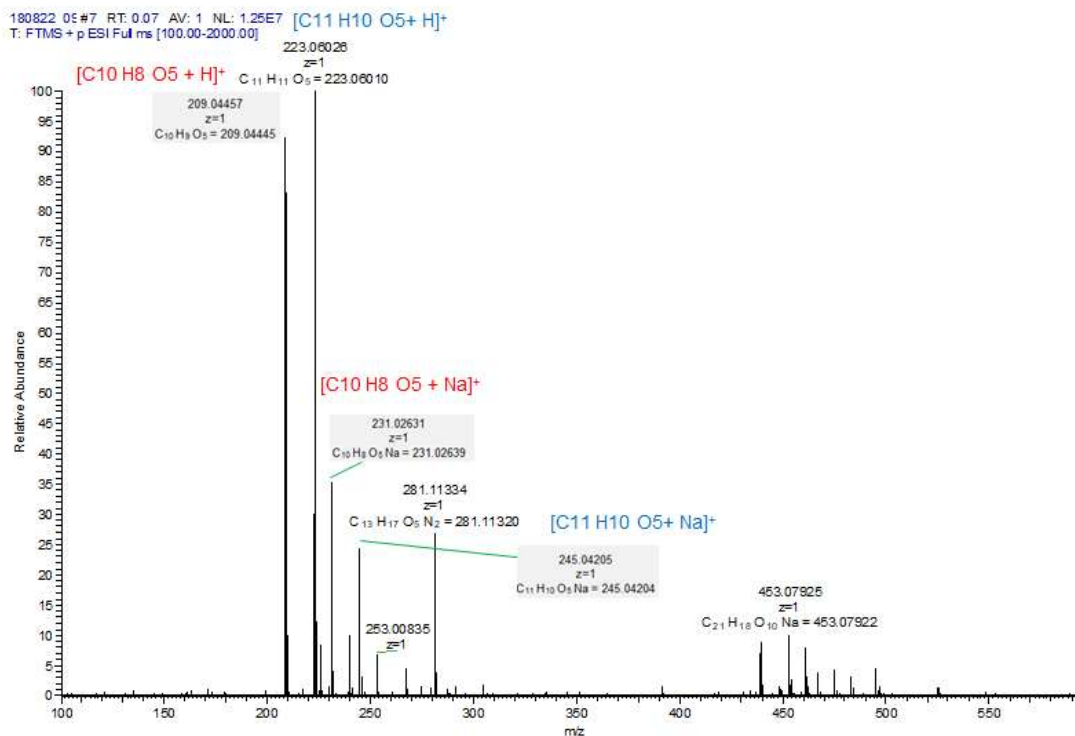

Supplement: Supplementary file 1 [file molecules-24-00889-s001.zip › Figure S11. ESI-MS spectrum of fraction 3.pdf]

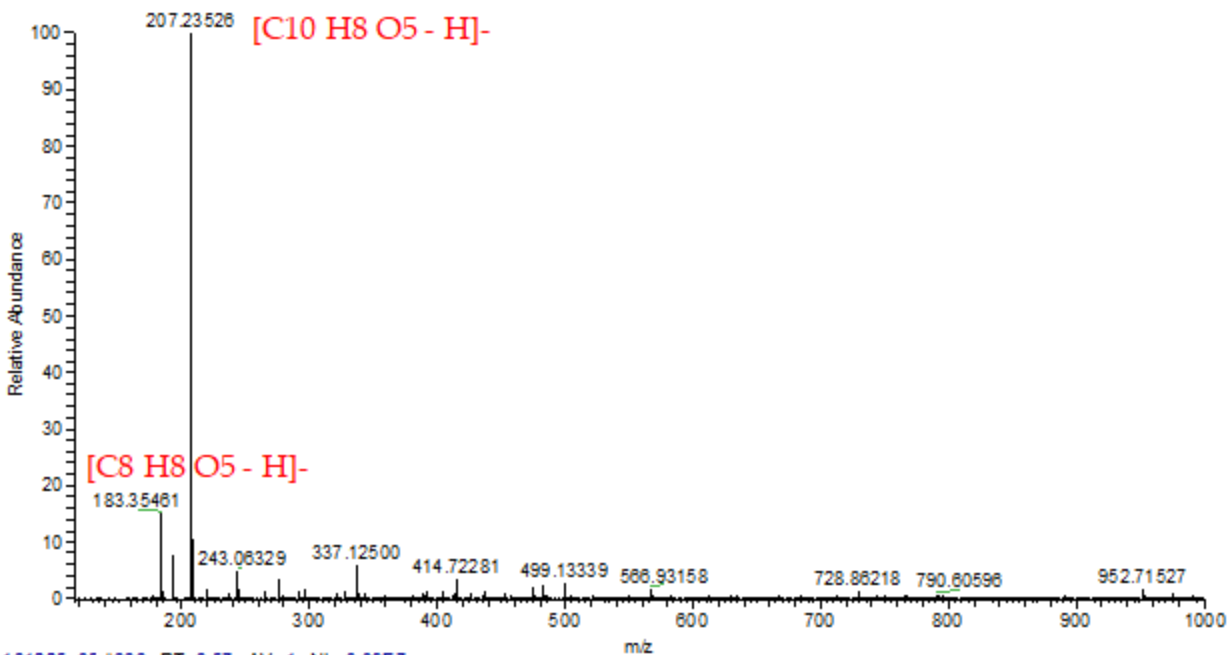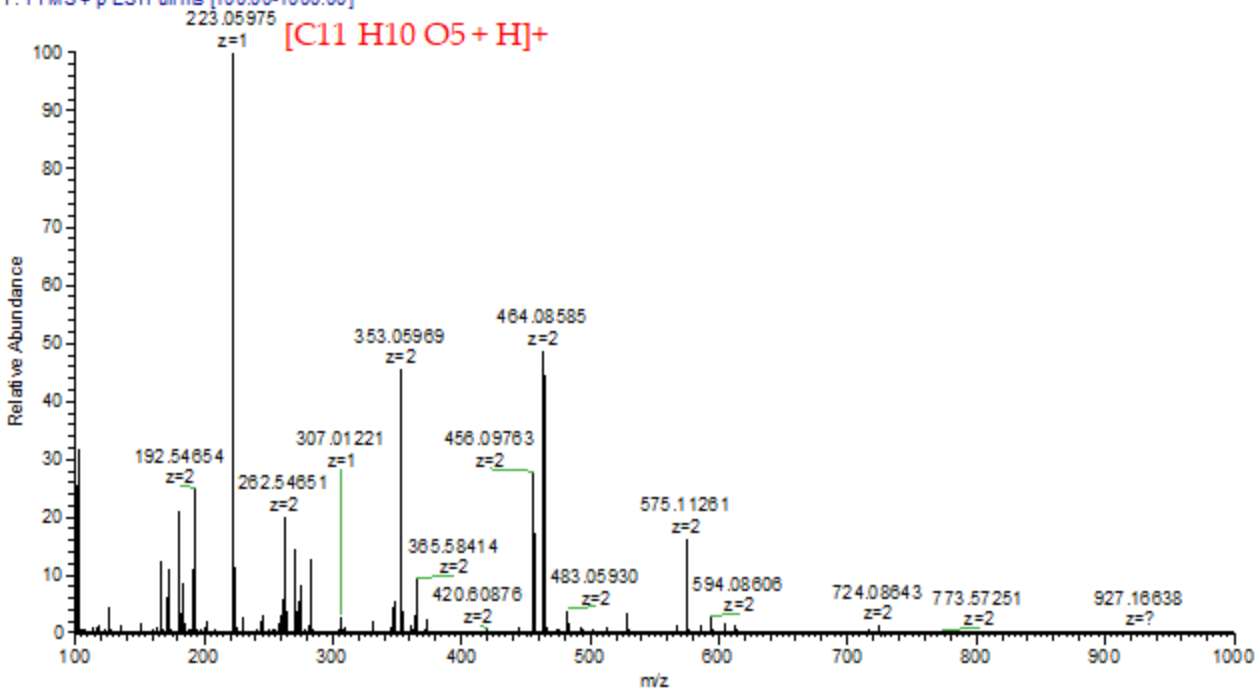

Supplement: Supplementary file 1 [file molecules-24-00889-s001.zip › Figure S12. LC-MS spectrum of fraction 3.pdf]

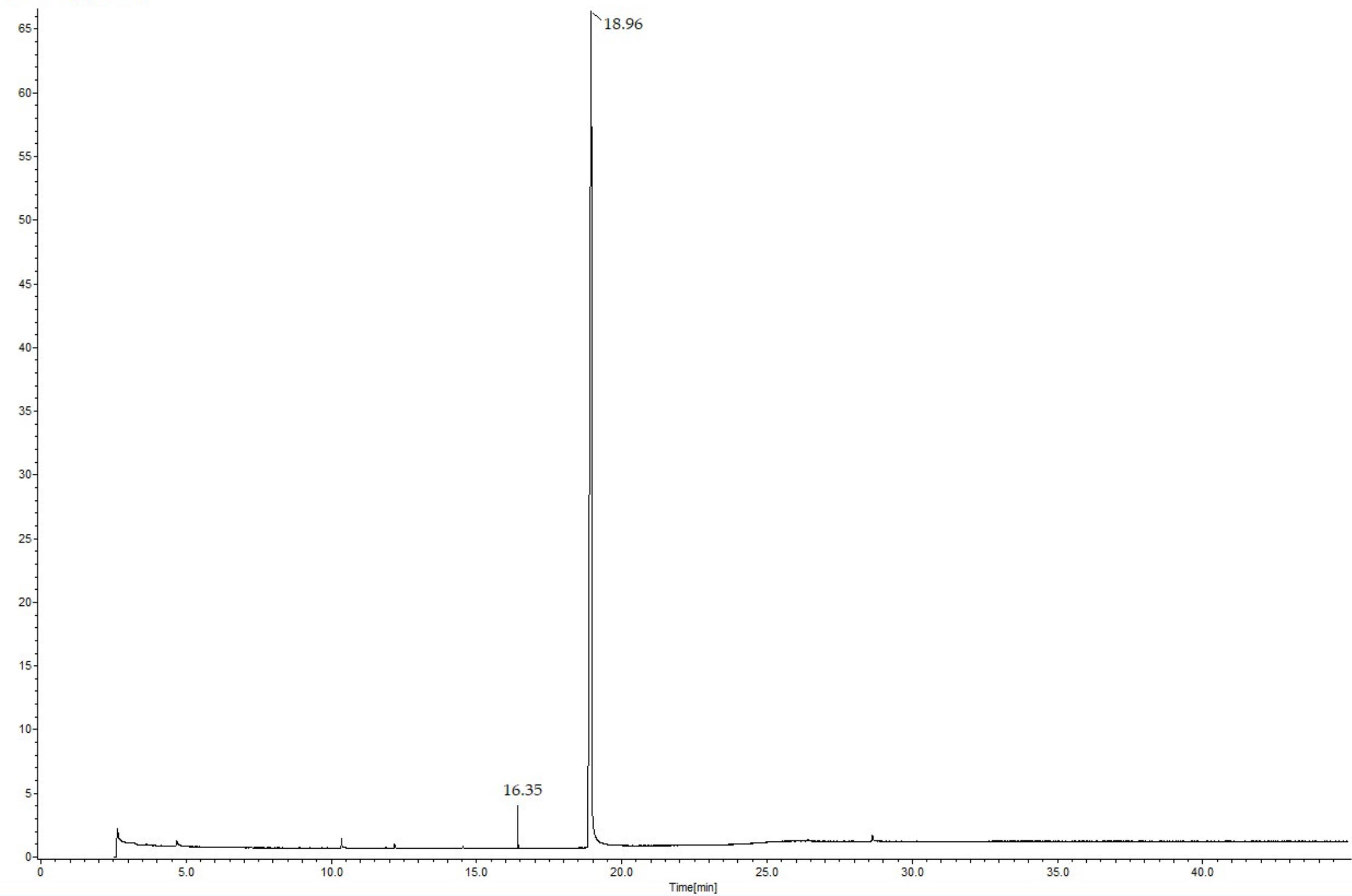

Supplement: Supplementary file 1 [file molecules-24-00889-s001.zip › Figure S13. GC-MS chromatogram of fraction 4.pdf]

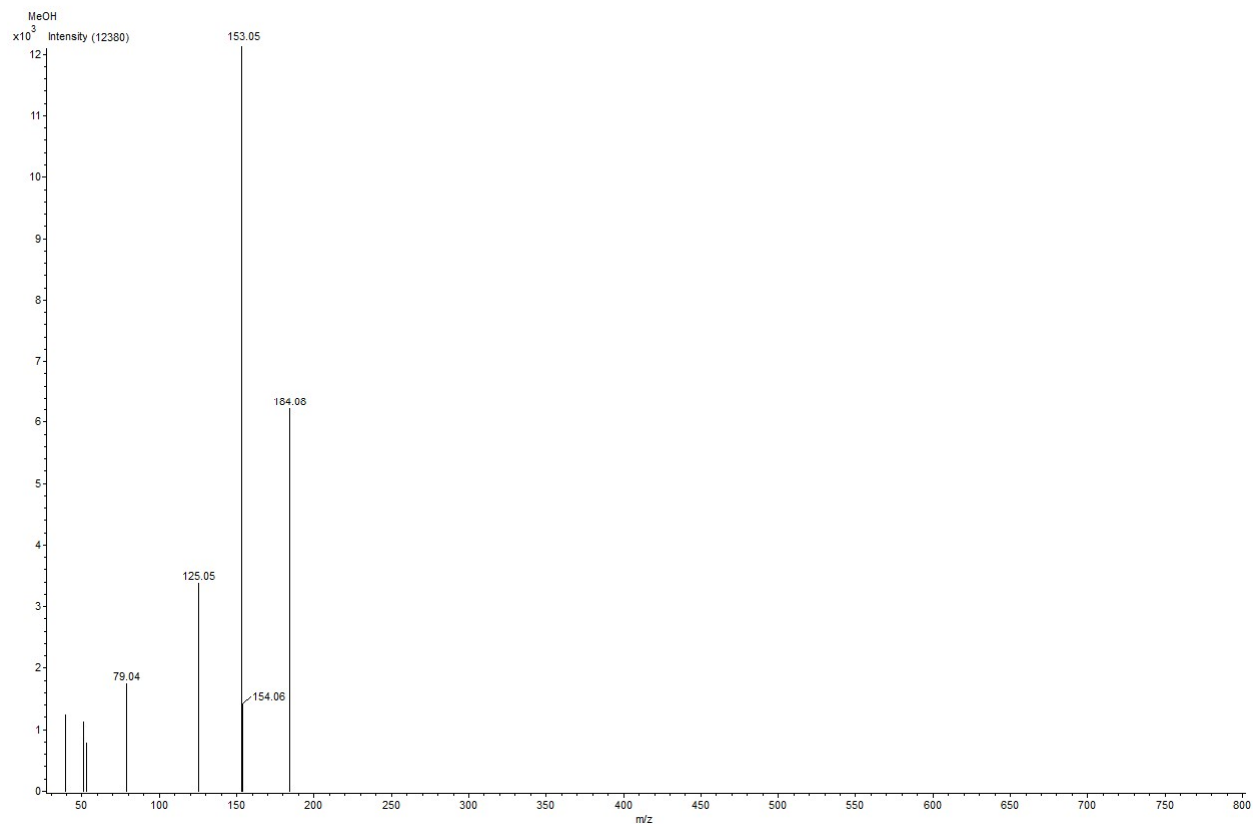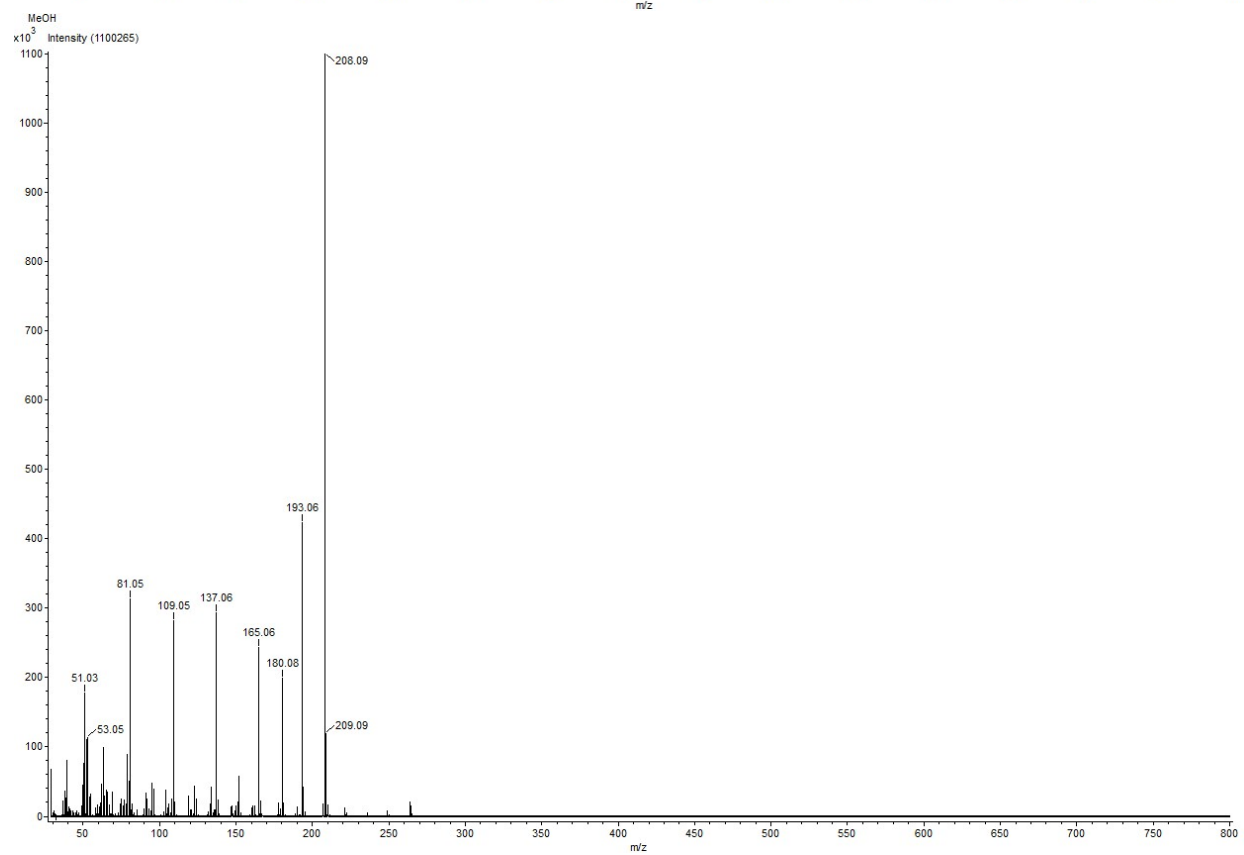

Supplement: Supplementary file 1 [file molecules-24-00889-s001.zip › Figure S14. GC-MS fragmentation of fraction 4.pdf]

# ESI-MS spectrum (Negative ion mode)

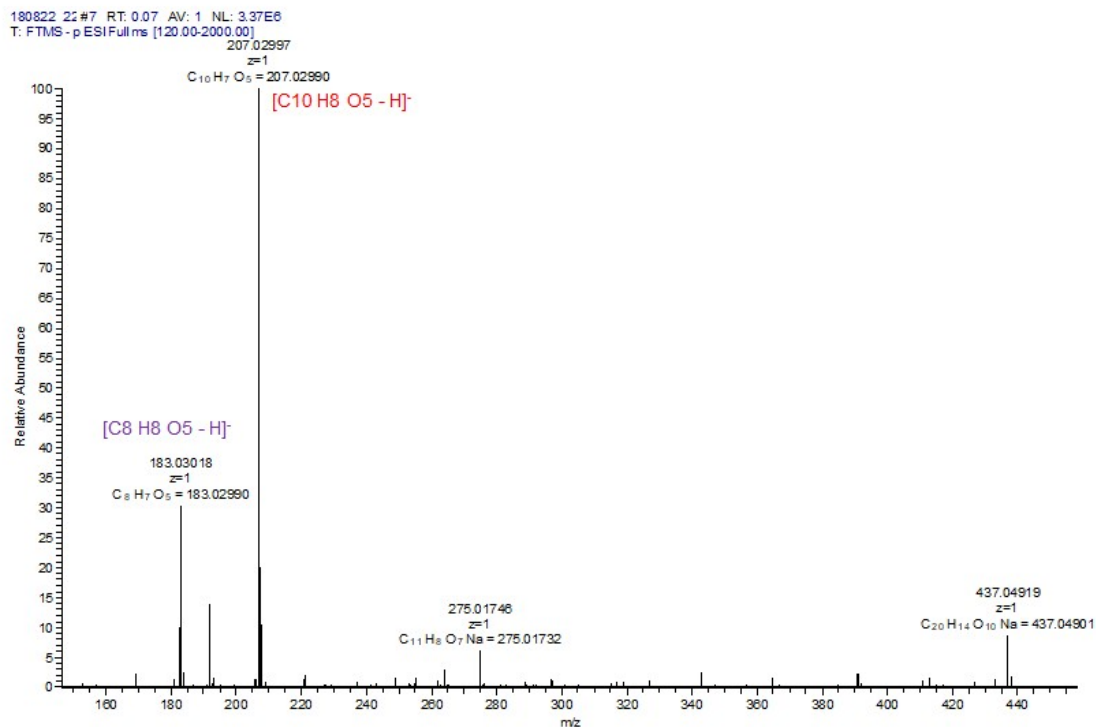

# ESI-MS spectrum (Positive ion mode)

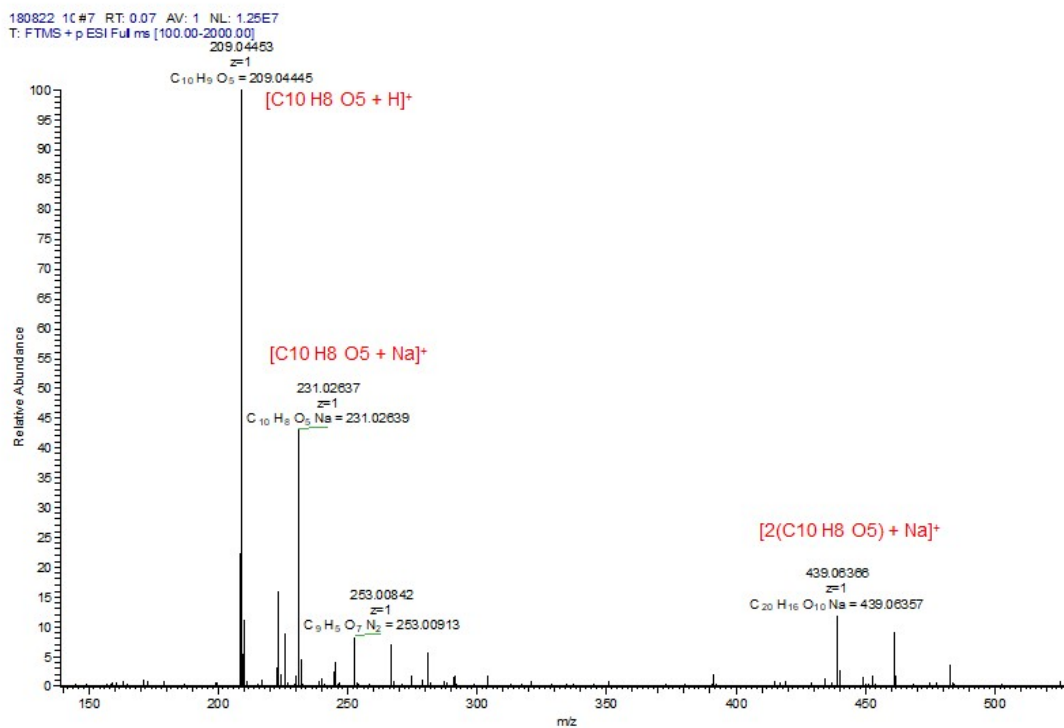

Supplement: Supplementary file 1 [file molecules-24-00889-s001.zip › Figure S15. ESI-MS spectrum of fraction 4.pdf]

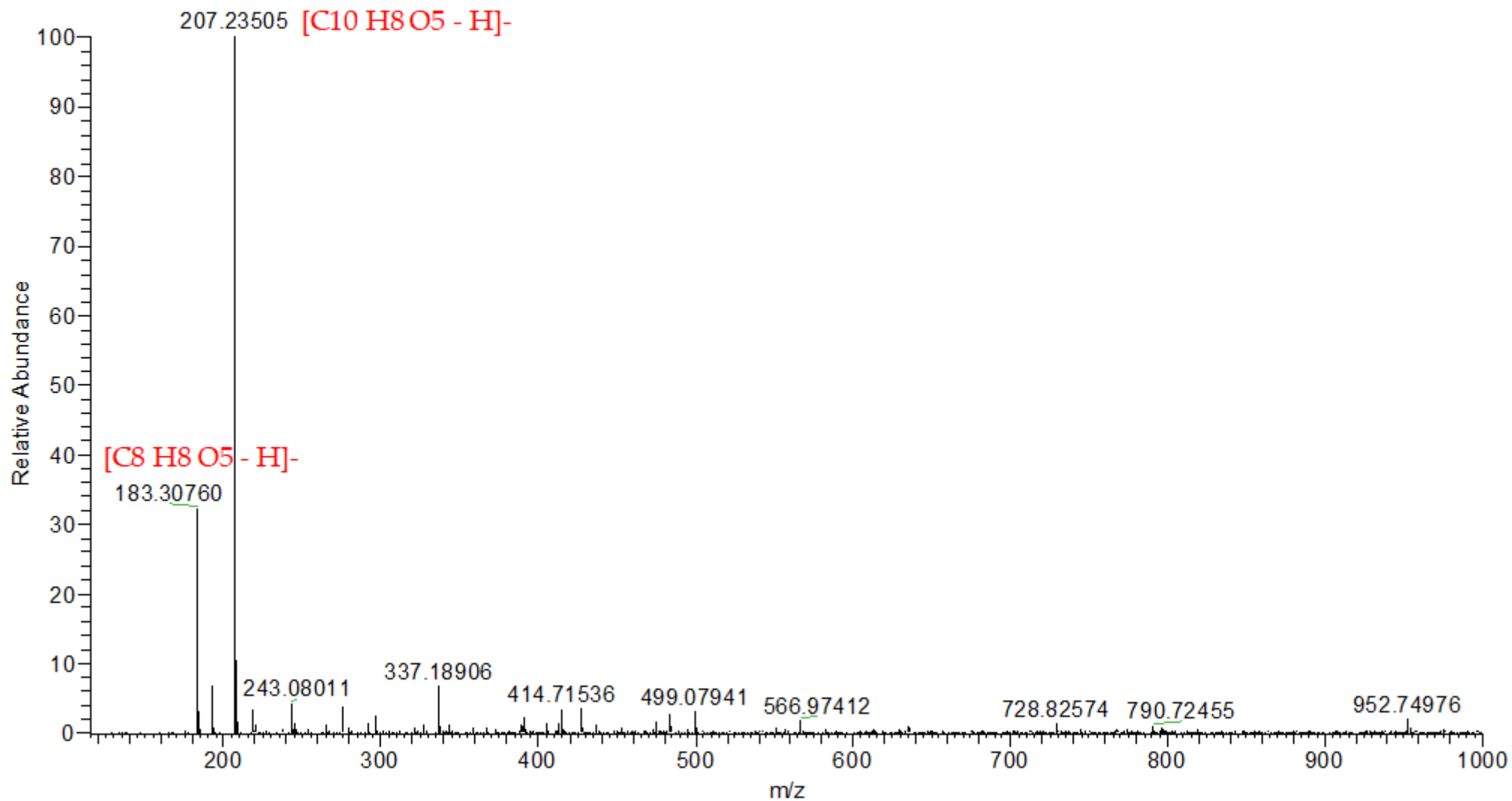

Supplement: Supplementary file 1 [file molecules-24-00889-s001.zip › Figure S16. LC-MS spectrum of fraction 4.pdf]

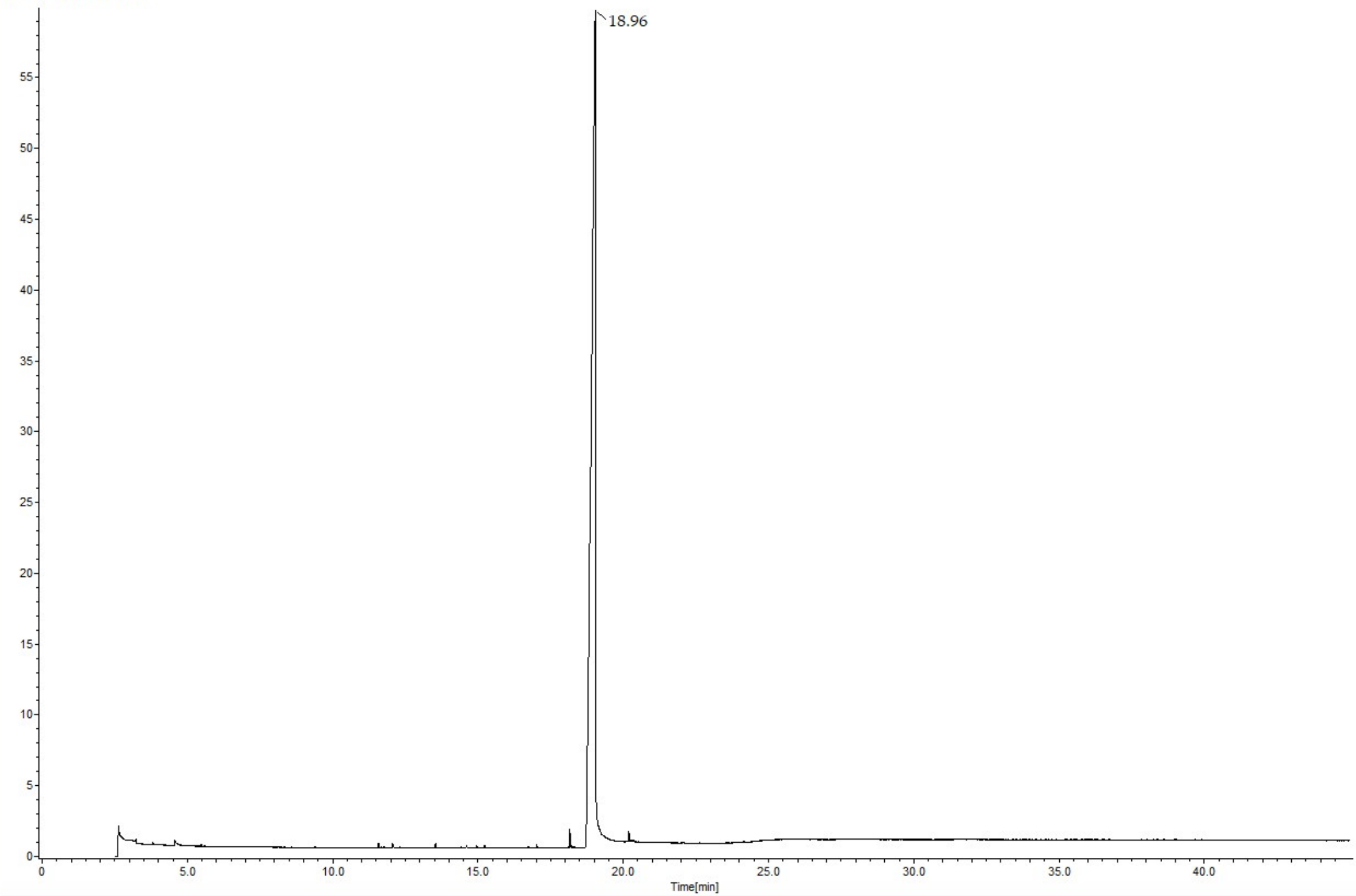

Supplement: Supplementary file 1 [file molecules-24-00889-s001.zip › Figure S17. GC-MS chromatogram of fraction 5.pdf]

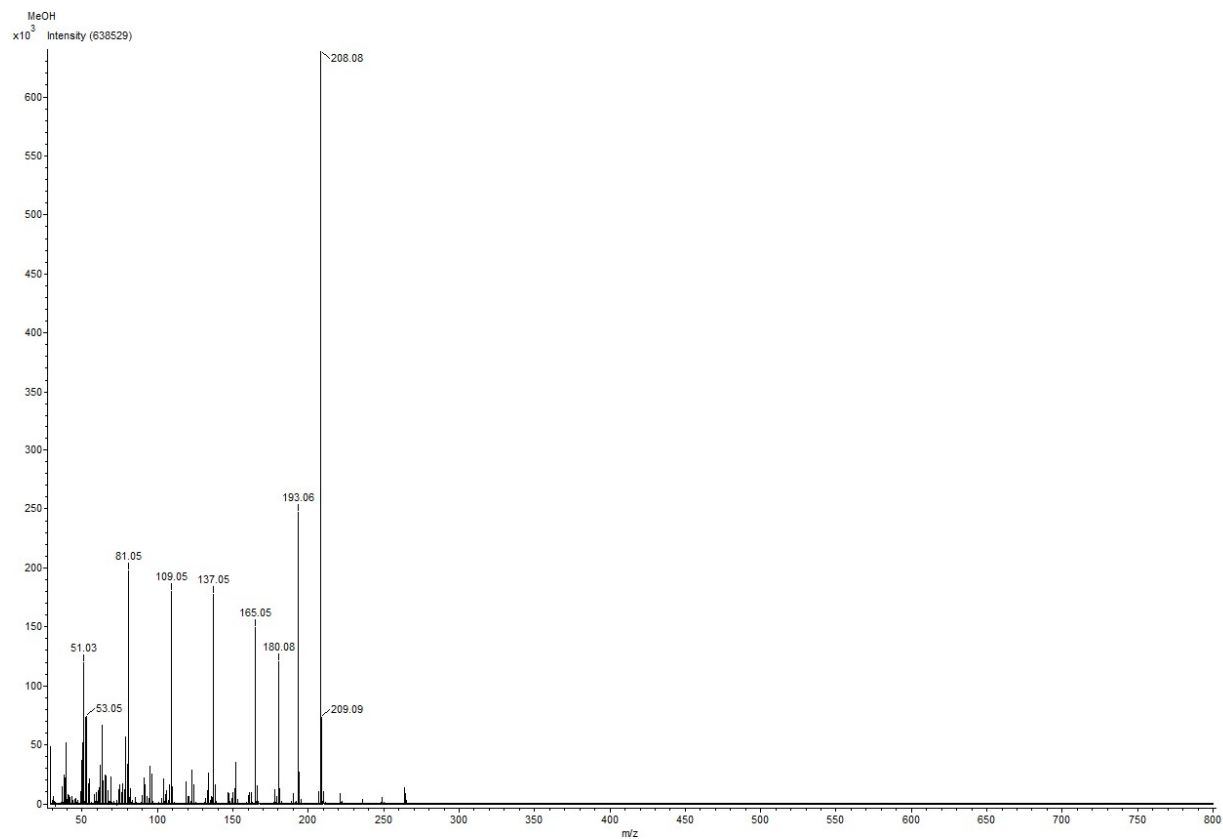

Supplement: Supplementary file 1 [file molecules-24-00889-s001.zip › Figure S18. GC-MS fragmentation of fraction 5.pdf]

## ESI-MS spectrum (Negative ion mode)

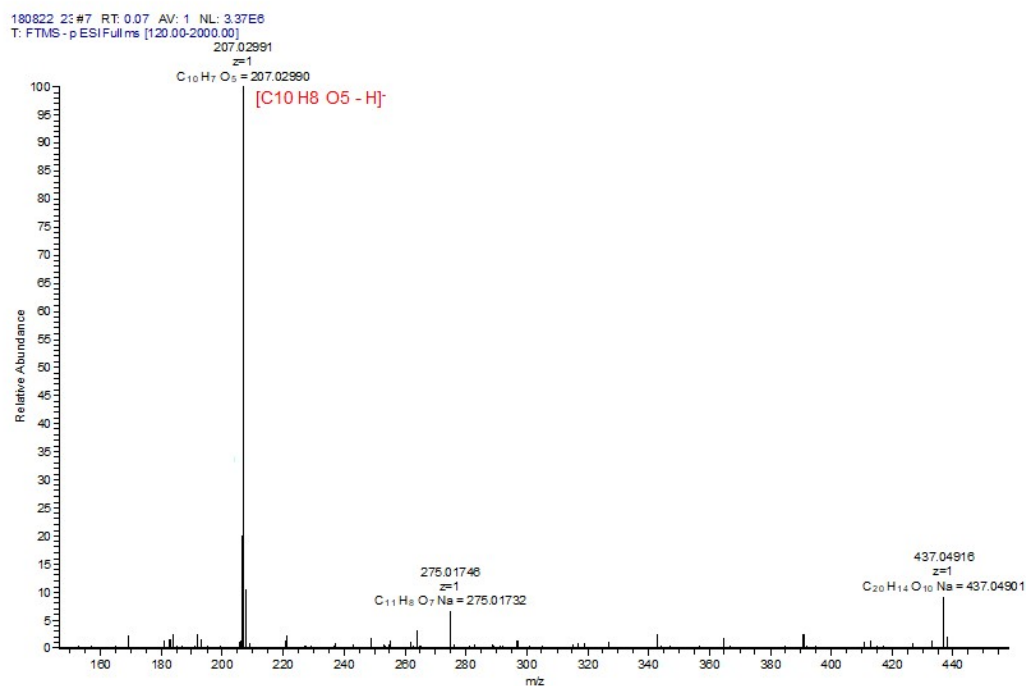

## ESI-MS spectrum (Positive ion mode)

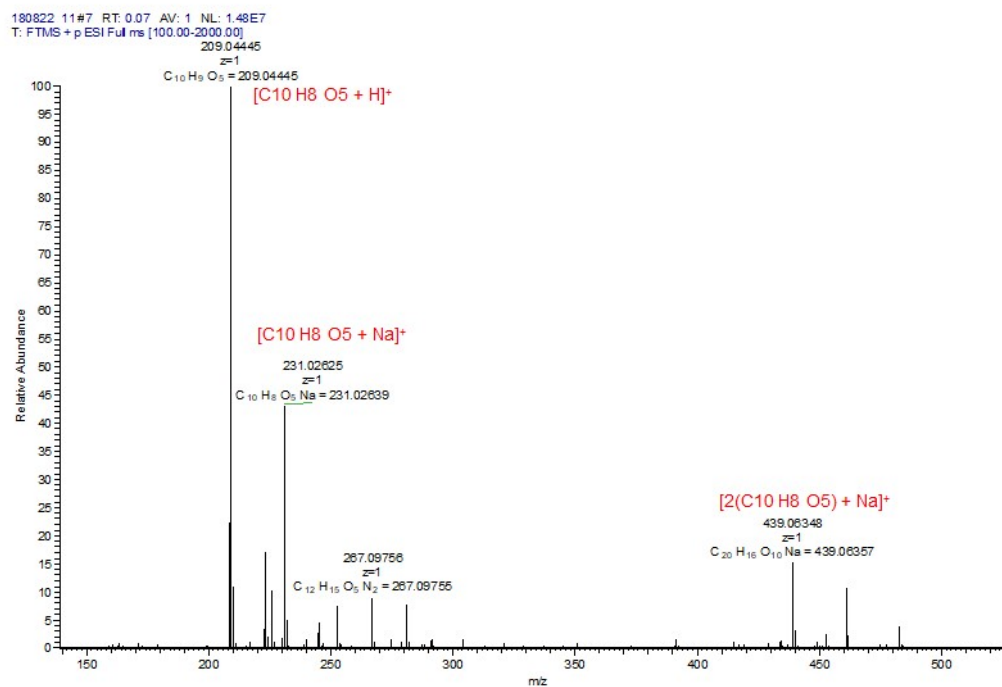

Supplement: Supplementary file 1 [file molecules-24-00889-s001.zip › Figure S19. ESI-MS spectrum of fraction 5.pdf]

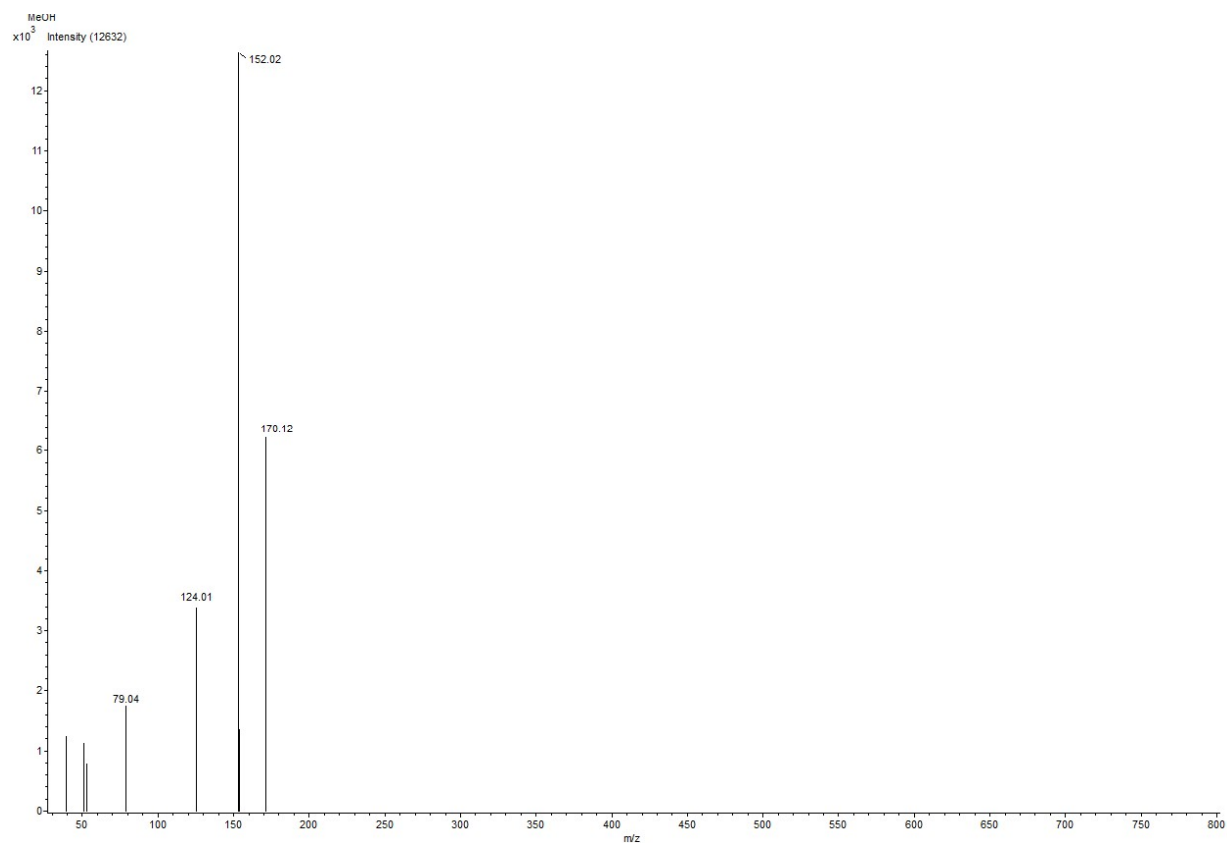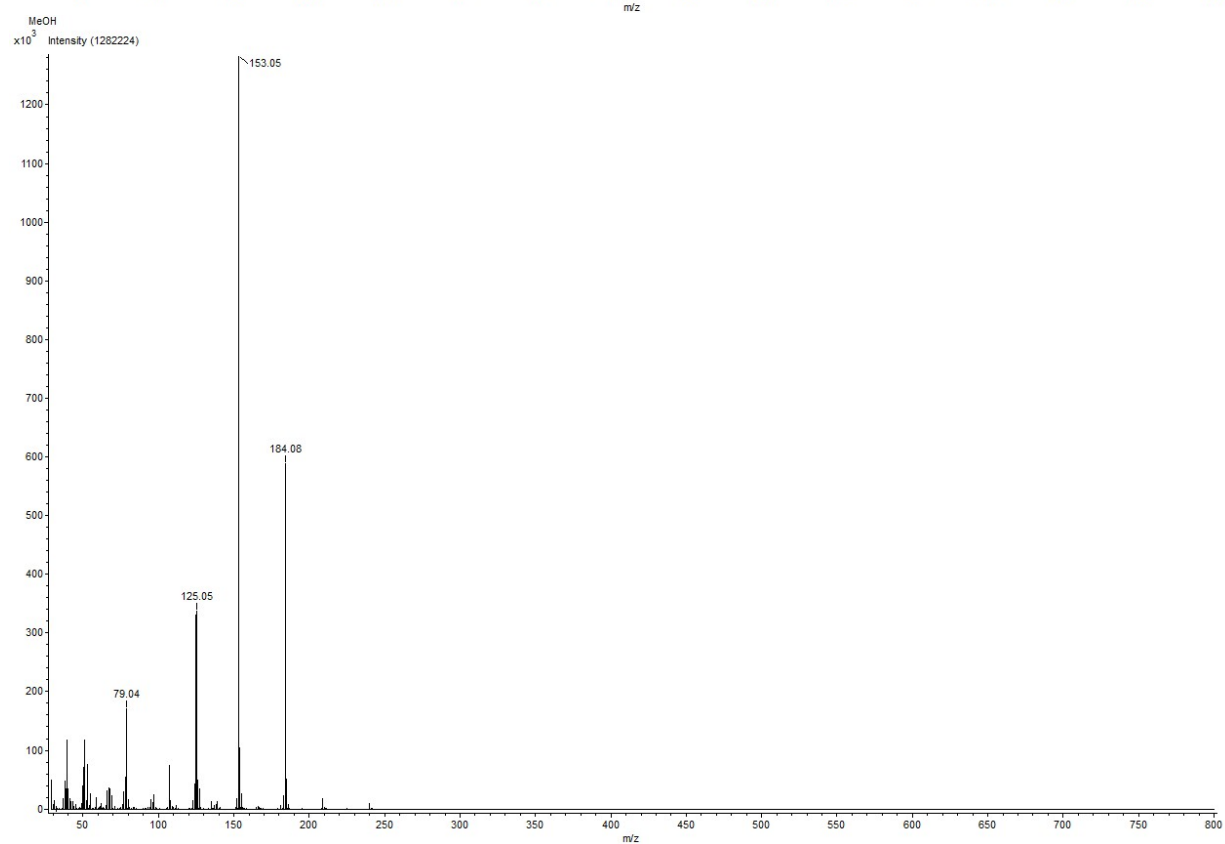

Supplement: Supplementary file 1 [file molecules-24-00889-s001.zip › Figure S2. GC-MS fragmentation of fraction 1.pdf]

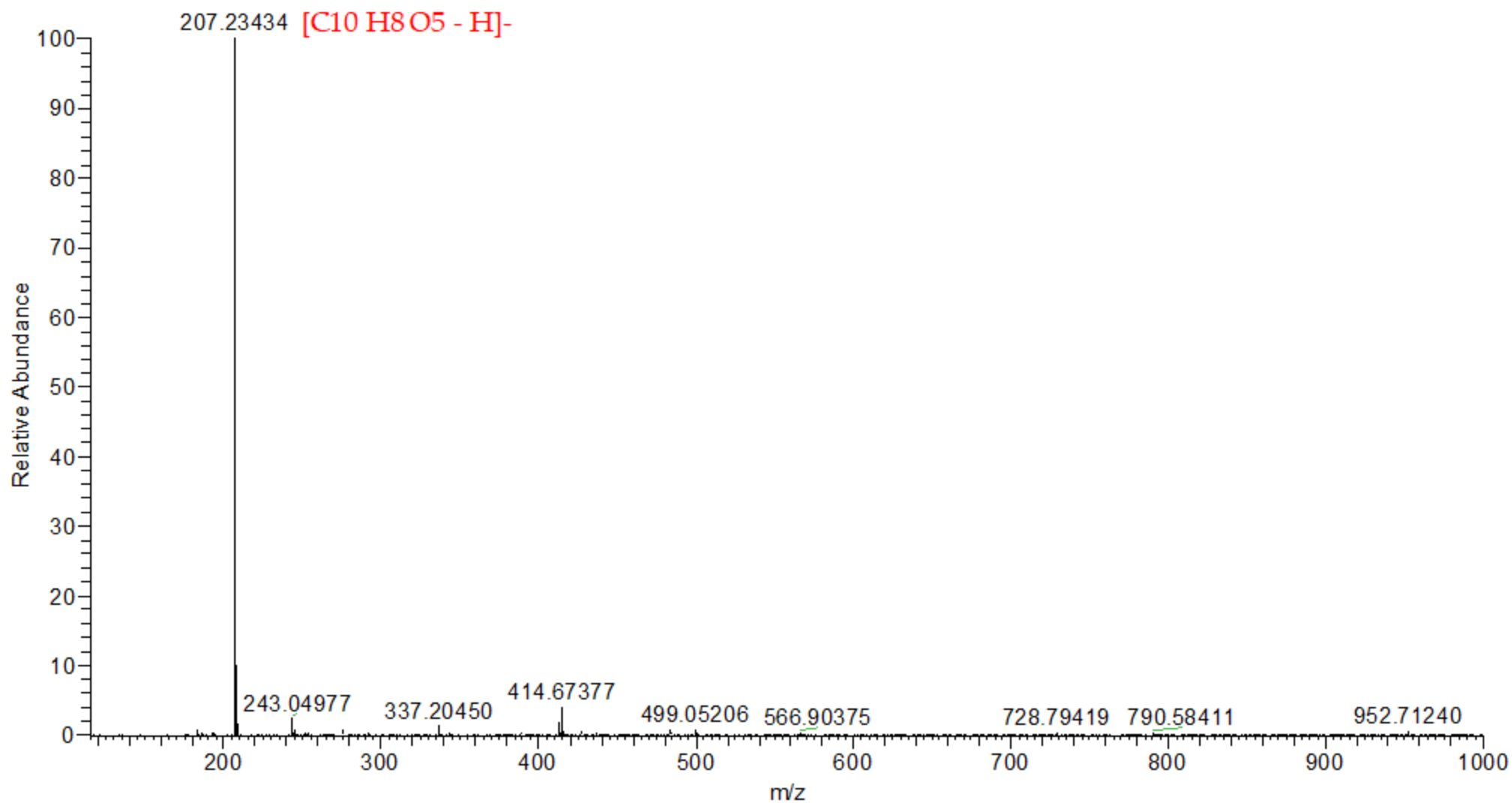

Supplement: Supplementary file 1 [file molecules-24-00889-s001.zip › Figure S20. LC-MS spectrum of fraction 5.pdf]

## TICs (total ion chromatograms) ITMS, Negative

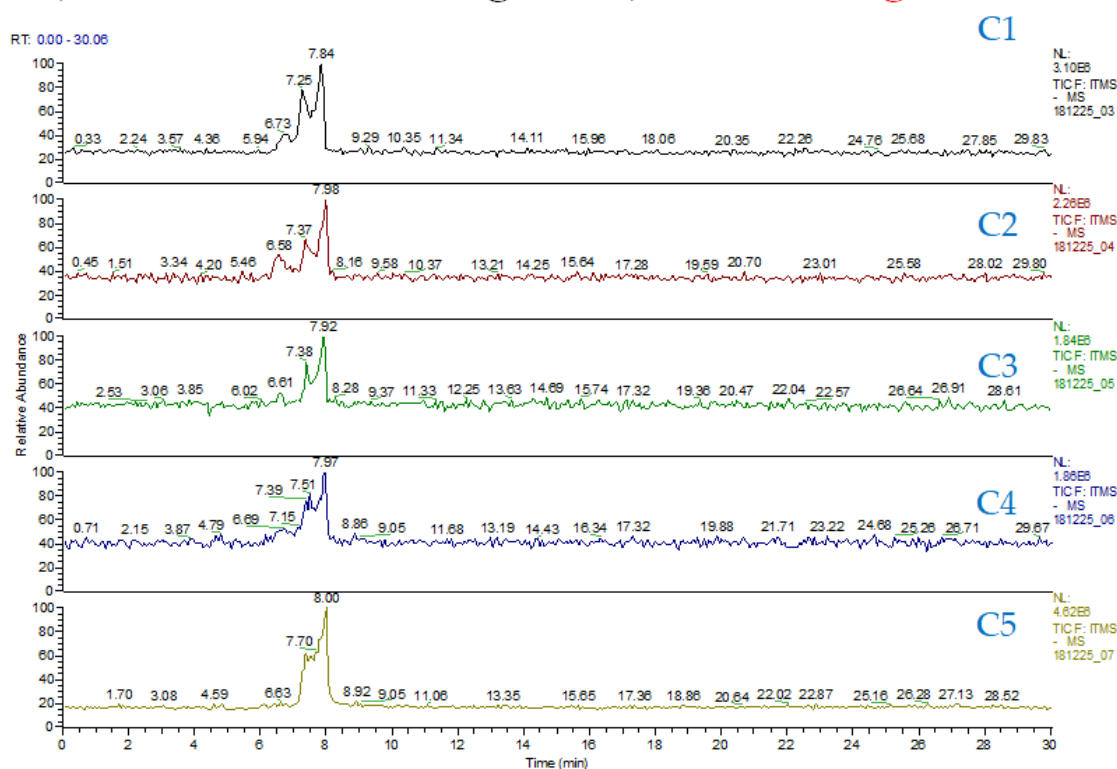

## TICs (total ion chromatograms) FTMS, Positive

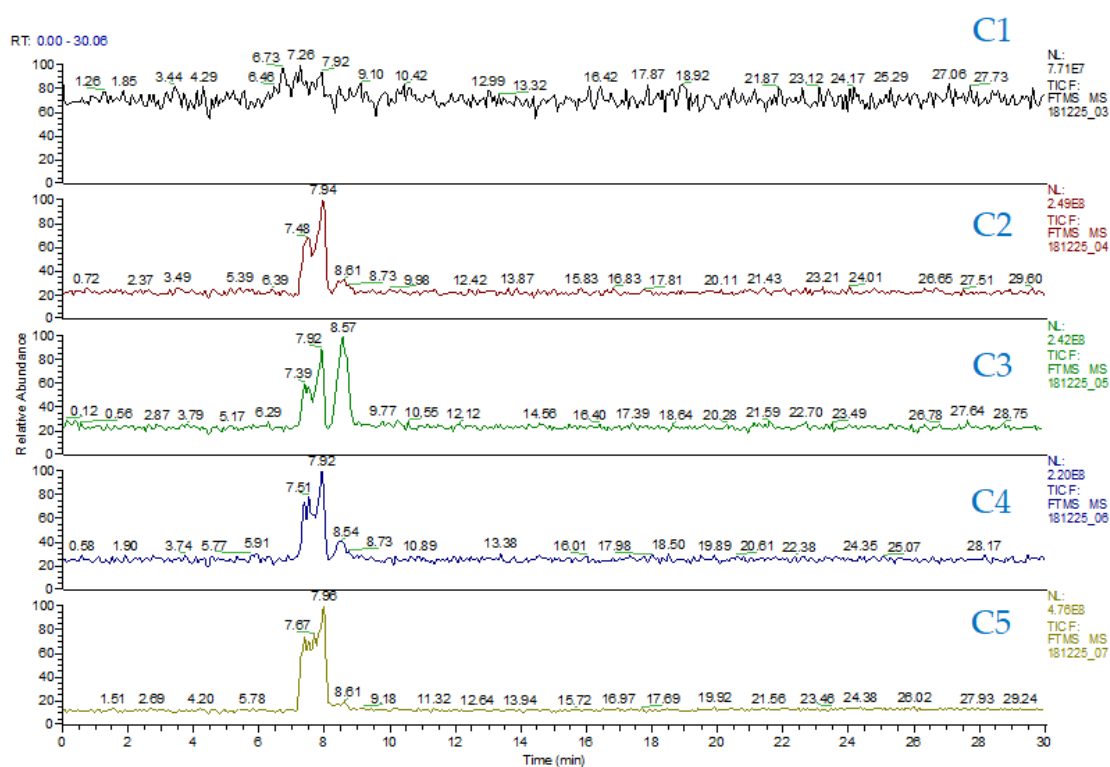

Supplement: Supplementary file 1 [file molecules-24-00889-s001.zip › Figure S21. LC-MS Total ion chromatograms of fractions 1-5.pdf]

181225\_03#470 RT: 6.20 AV: 1 NL: 1.24E2  
T: ITMS - c ESI Full ms [115.00-1000.00]

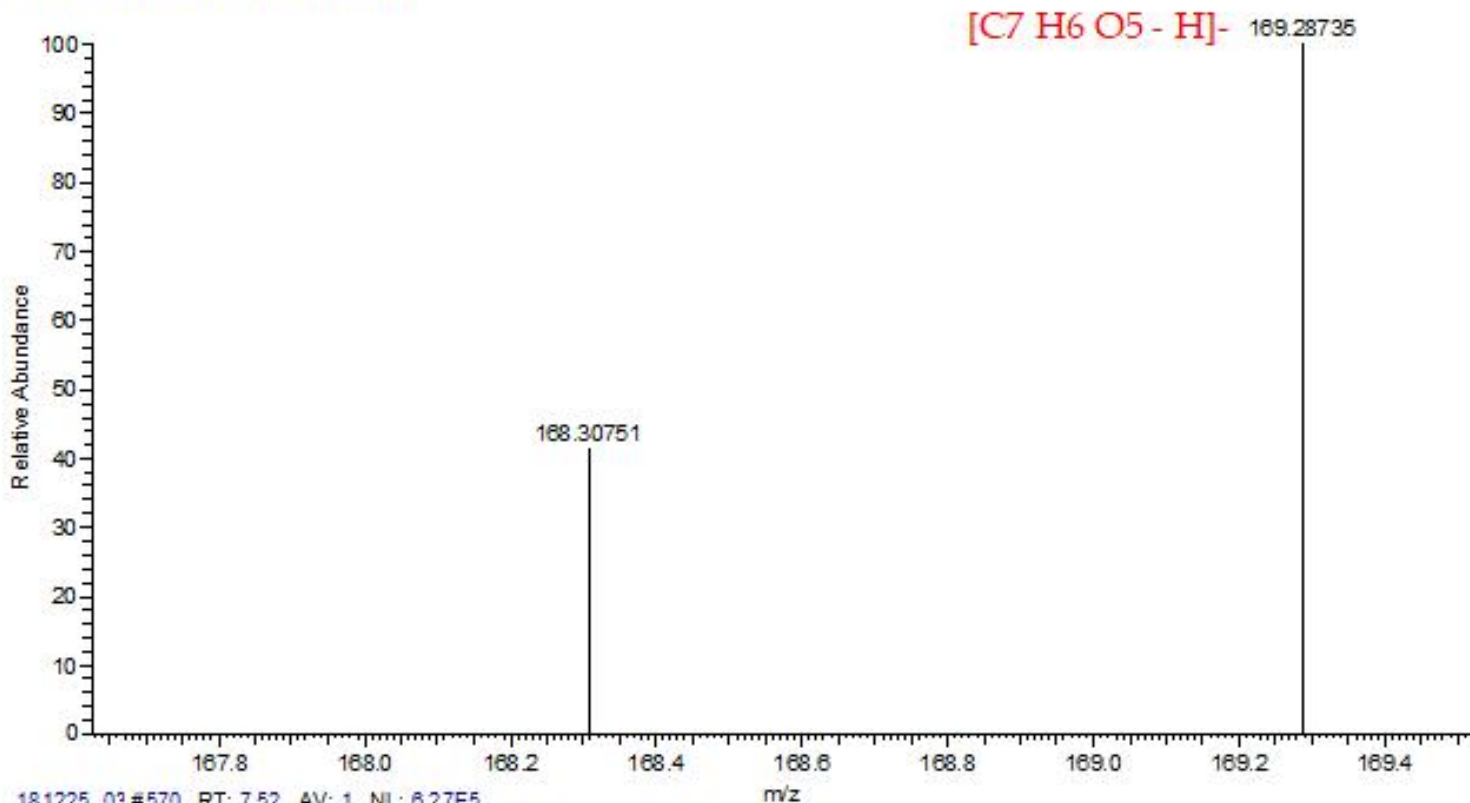

181225\_03#570 RT: 7.52 AV: 1 NL: 6.27E5  
T: ITMS - c ESI Full ms [115.00-1000.00]

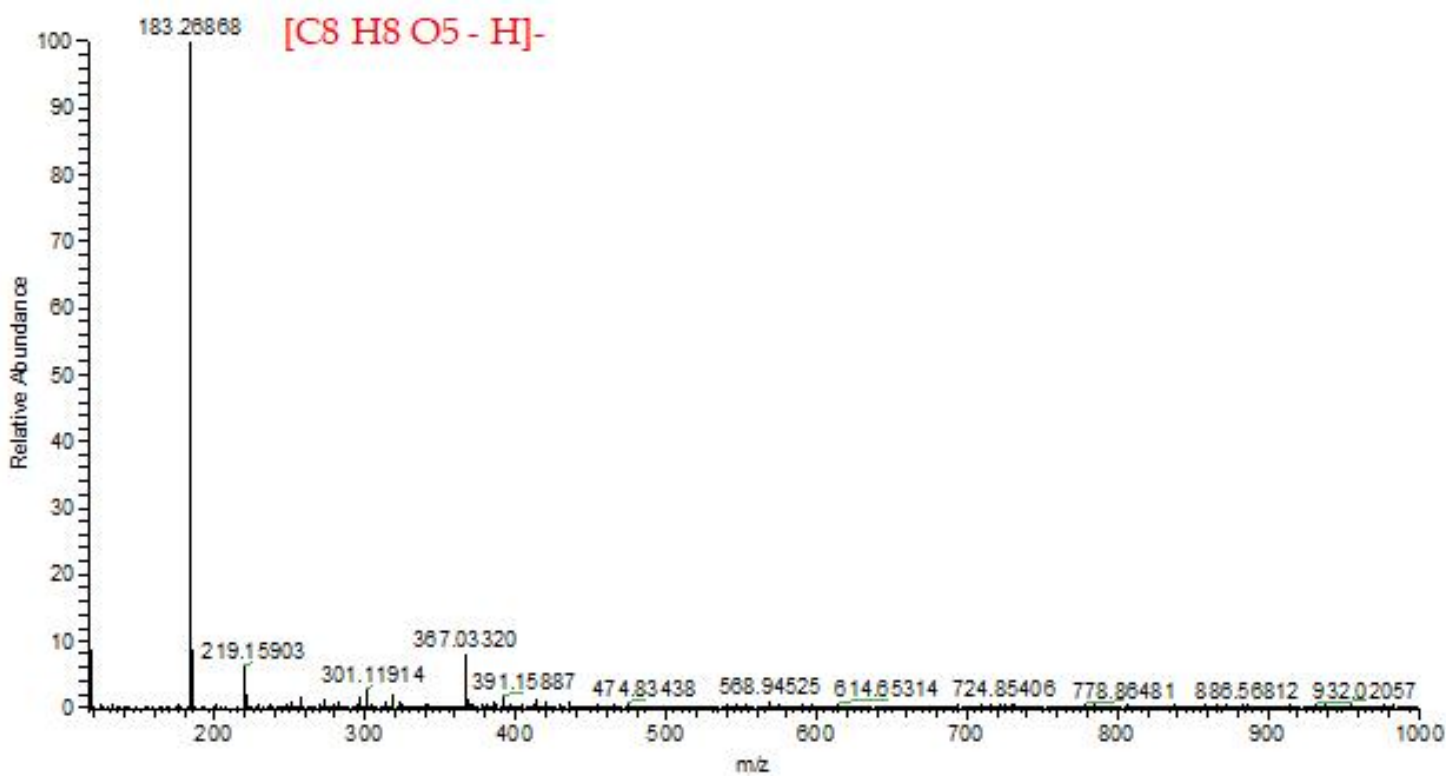

Supplement: Supplementary file 1 [file molecules-24-00889-s001.zip › Figure S4. LC-MS spectrum of fraction 1.pdf]

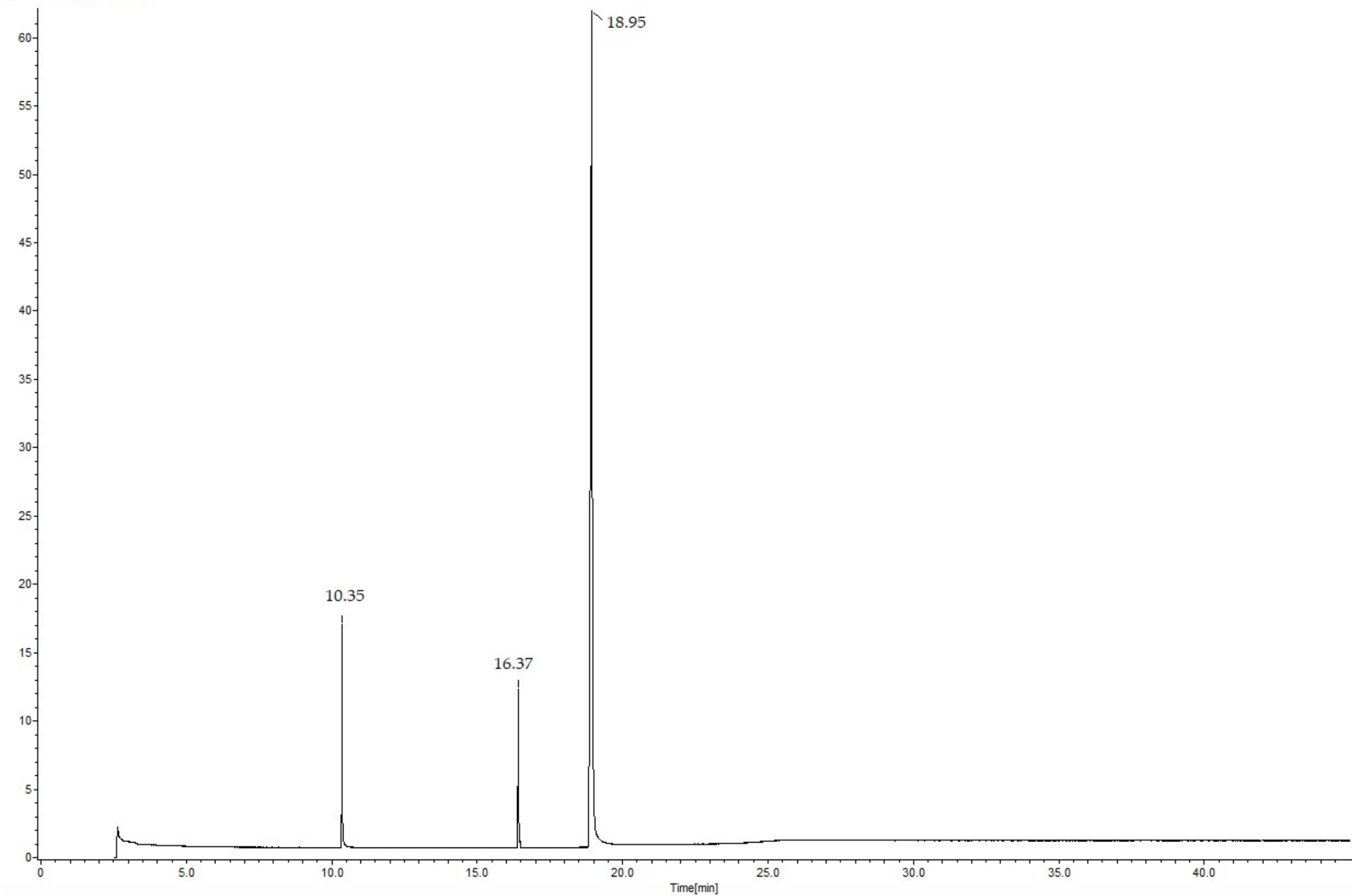

Supplement: Supplementary file 1 [file molecules-24-00889-s001.zip › Figure S5. GC-MS chromatogram of fraction 2.pdf]

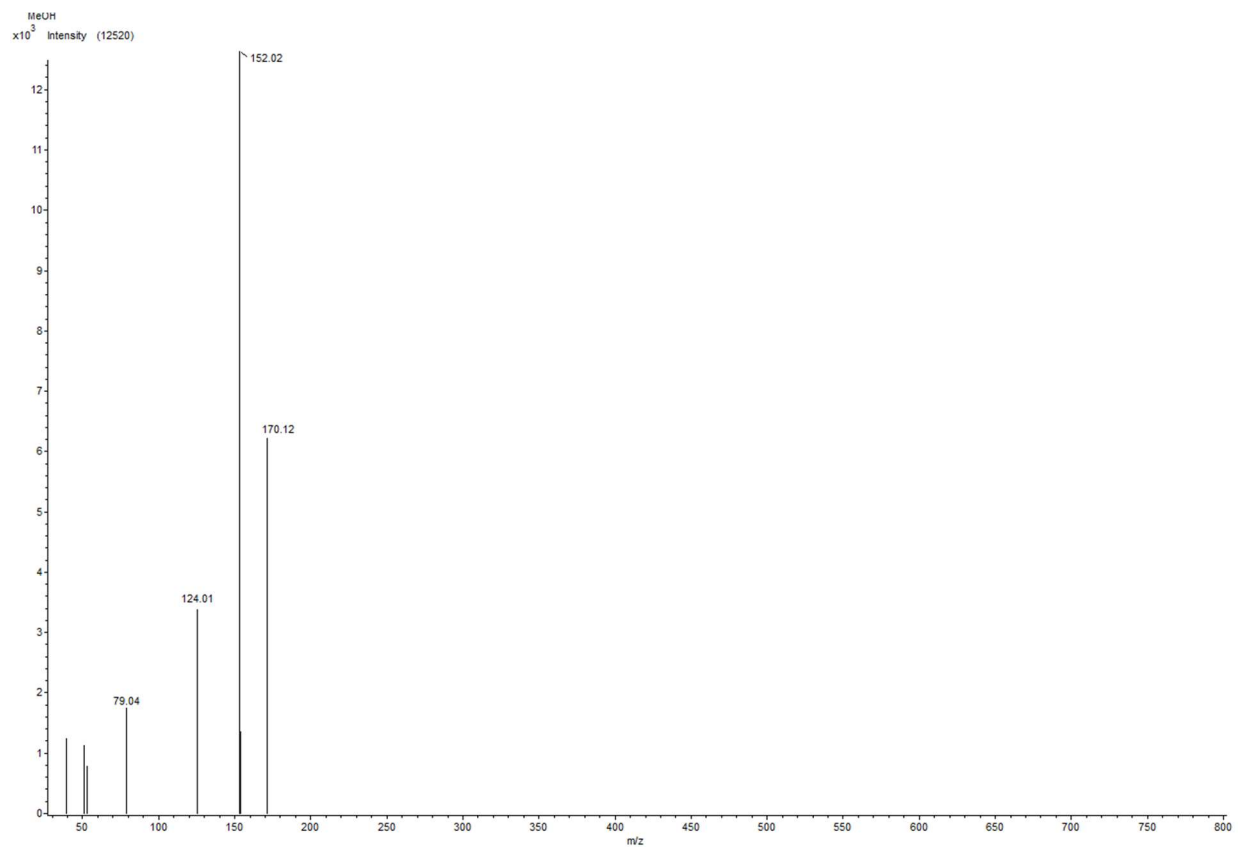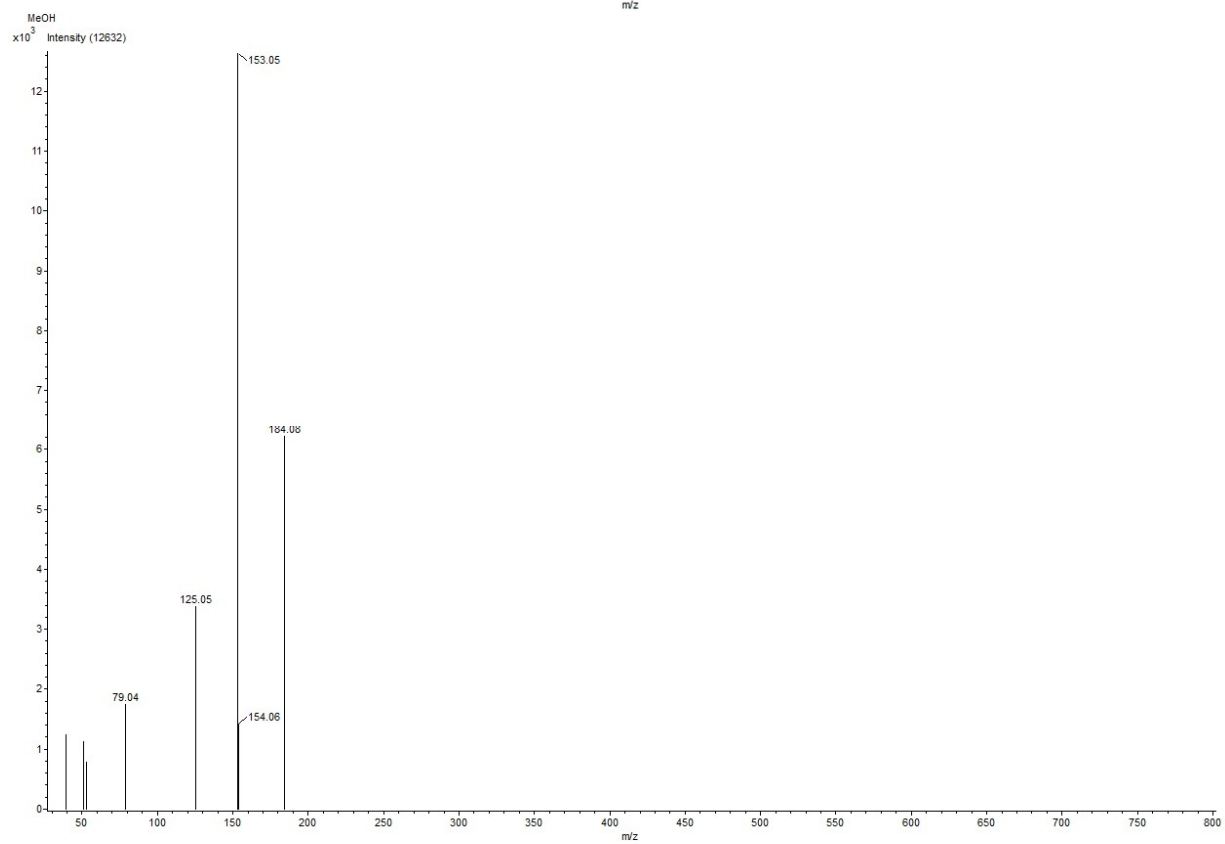

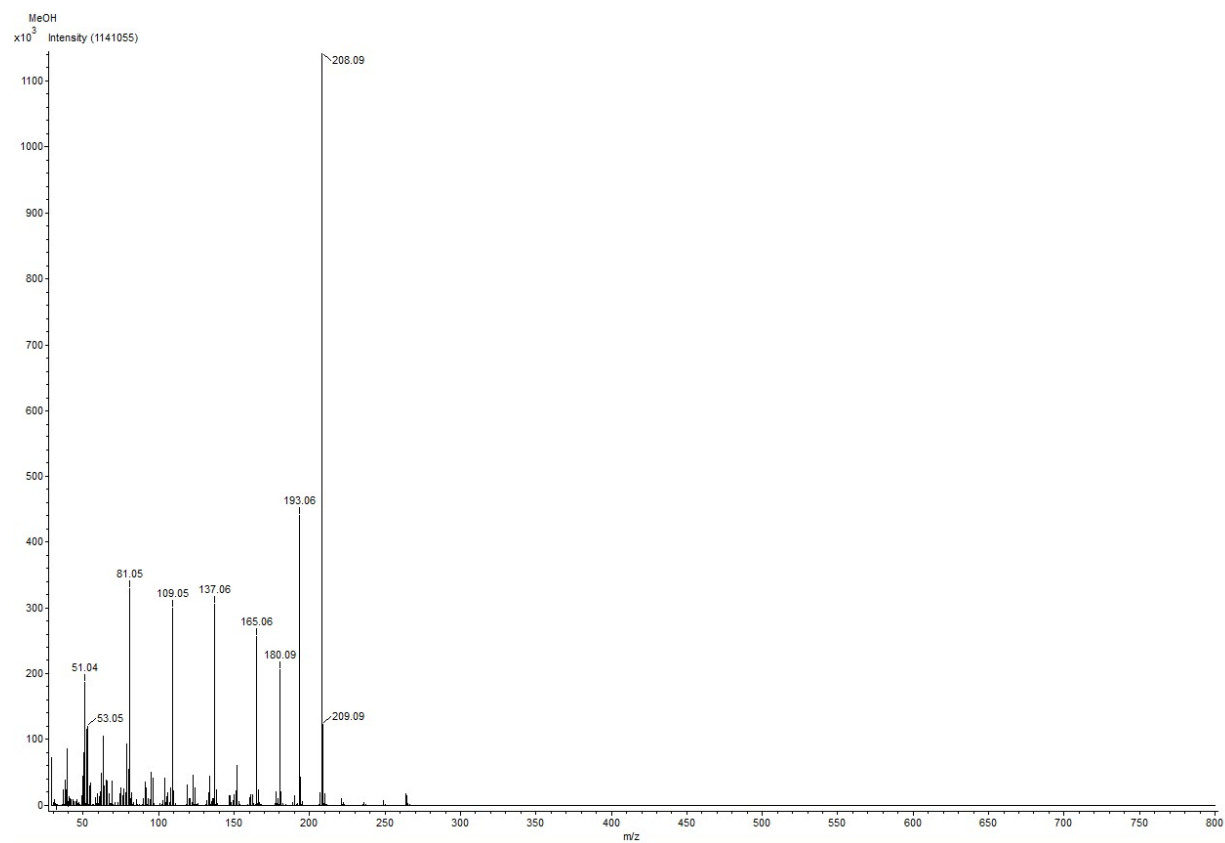

Supplement: Supplementary file 1 [file molecules-24-00889-s001.zip › Figure S6 GC-MS fragmentation of fraction 2.pdf]

## ESI-MS spectrum (Negative ion mode)

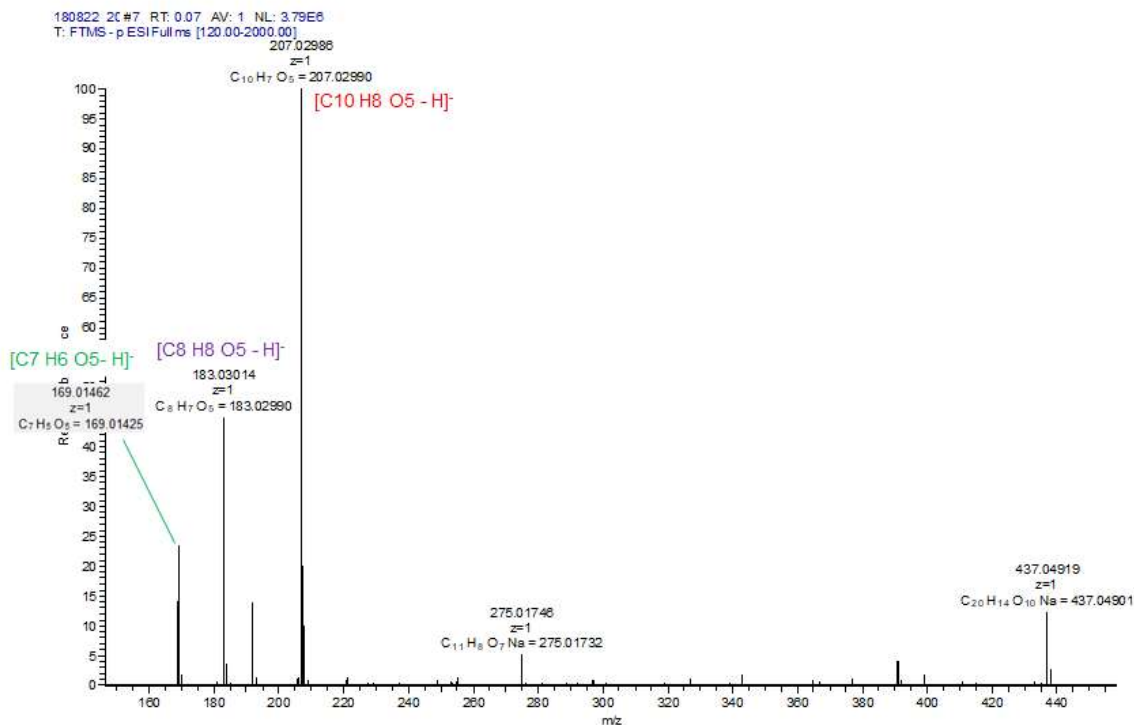

## ESI-MS spectrum (Positive ion mode)

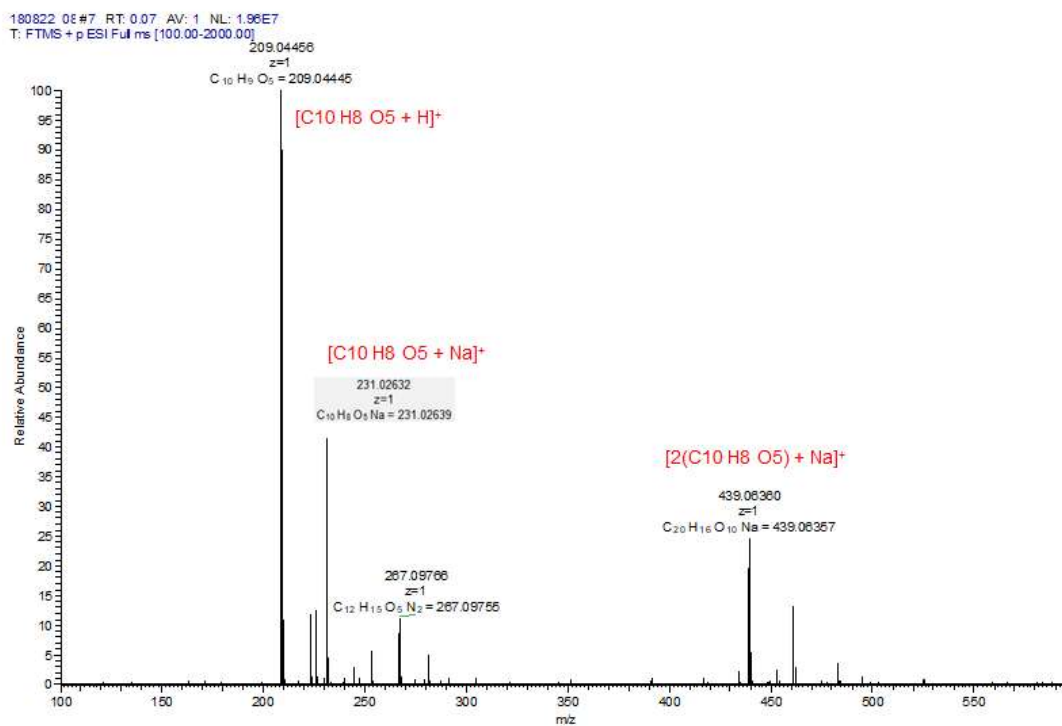

Supplement: Supplementary file 1 [file molecules-24-00889-s001.zip › Figure S7. ESI-MS spectrum of fraction 2.pdf]

181225\_04#610 RT: 7.93 AV: 1 NL: 6.64E5  
T: ITMS - c ESI Full ms [115.00-1000.00]

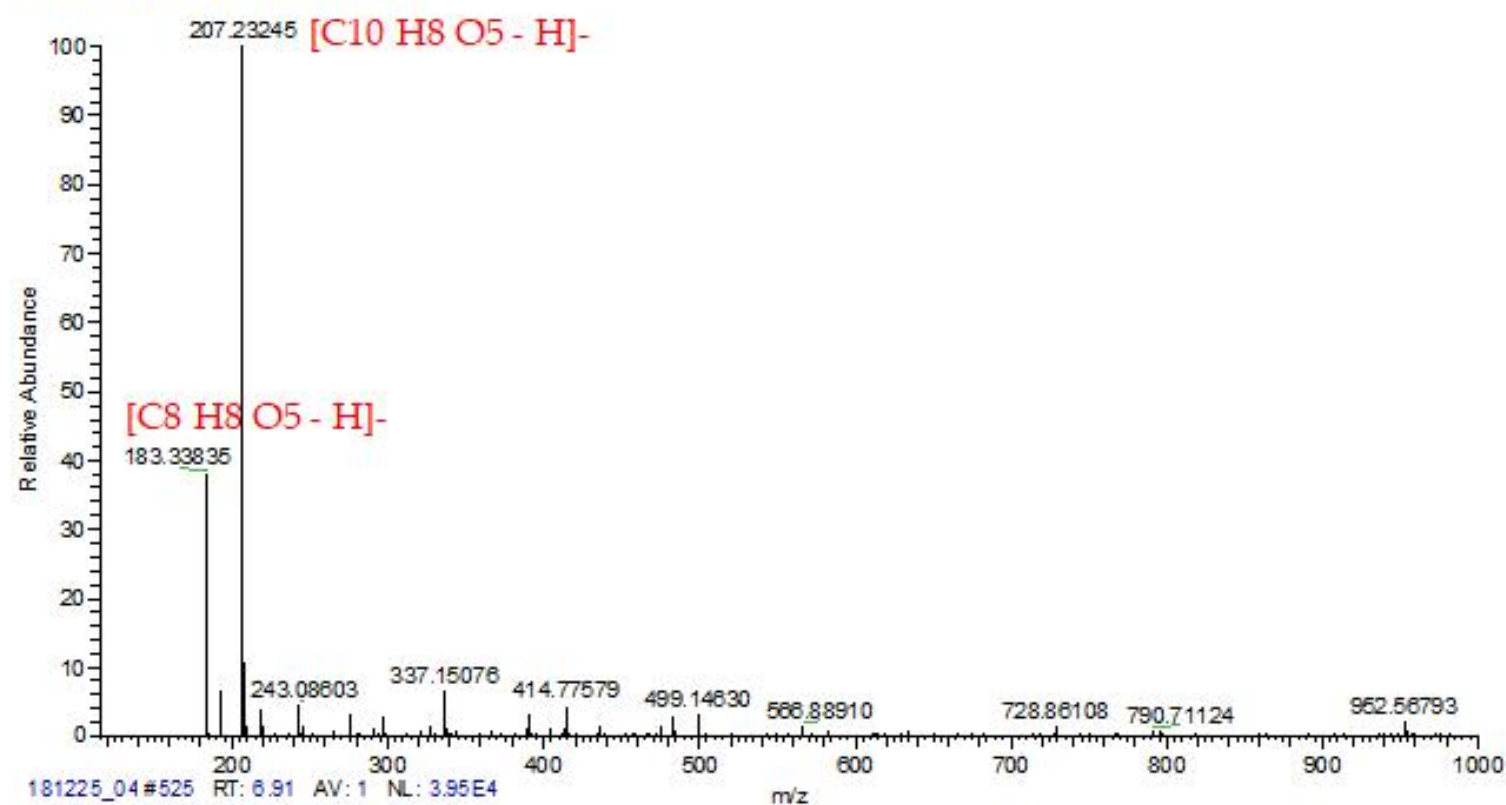

181225\_04#525 RT: 6.91 AV: 1 NL: 3.95E4  
T: ITMS - c ESI Full ms [115.00-1000.00]

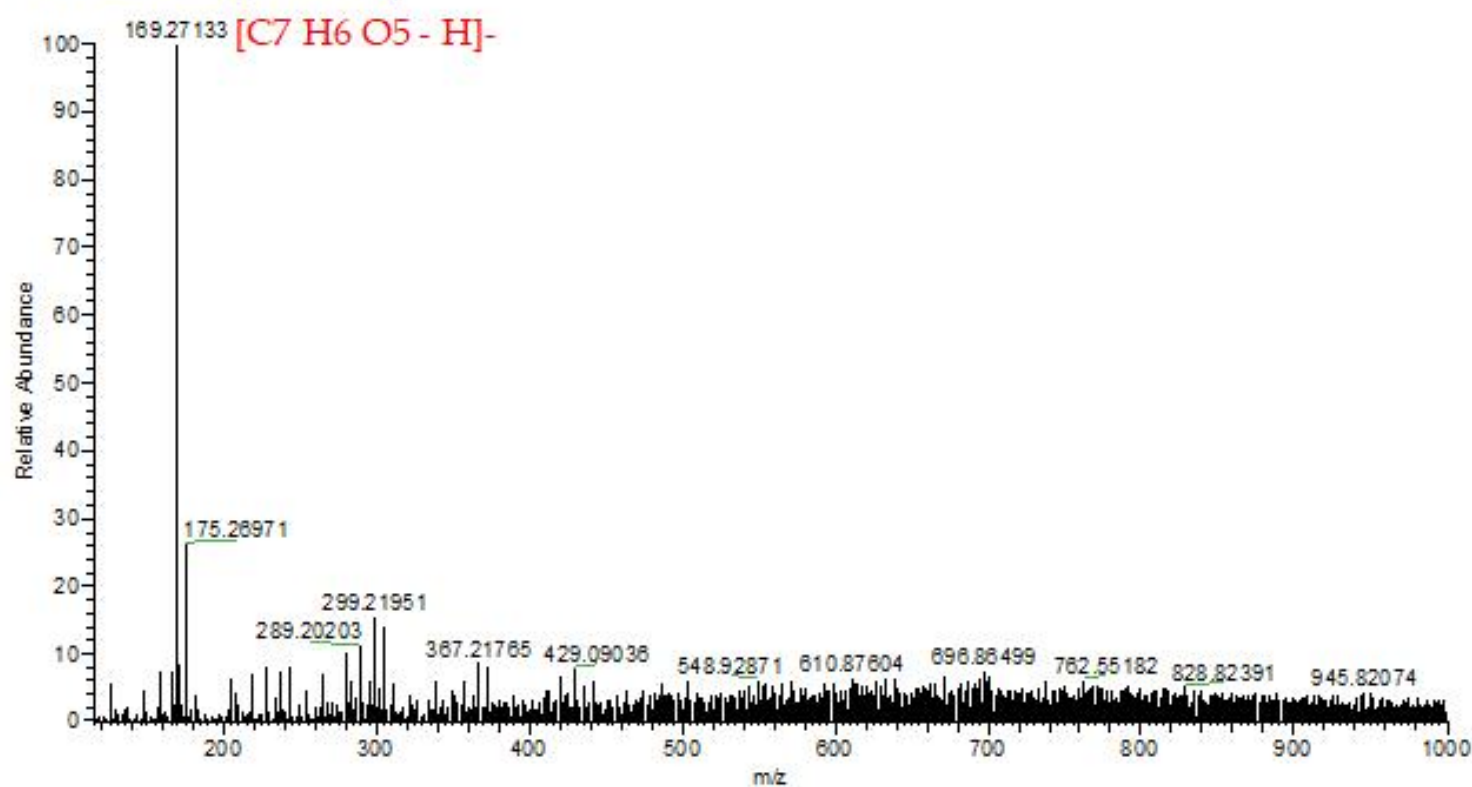

Supplement: Supplementary file 1 [file molecules-24-00889-s001.zip › Figure S8. LC-MS spectrum of fraction 2.pdf]

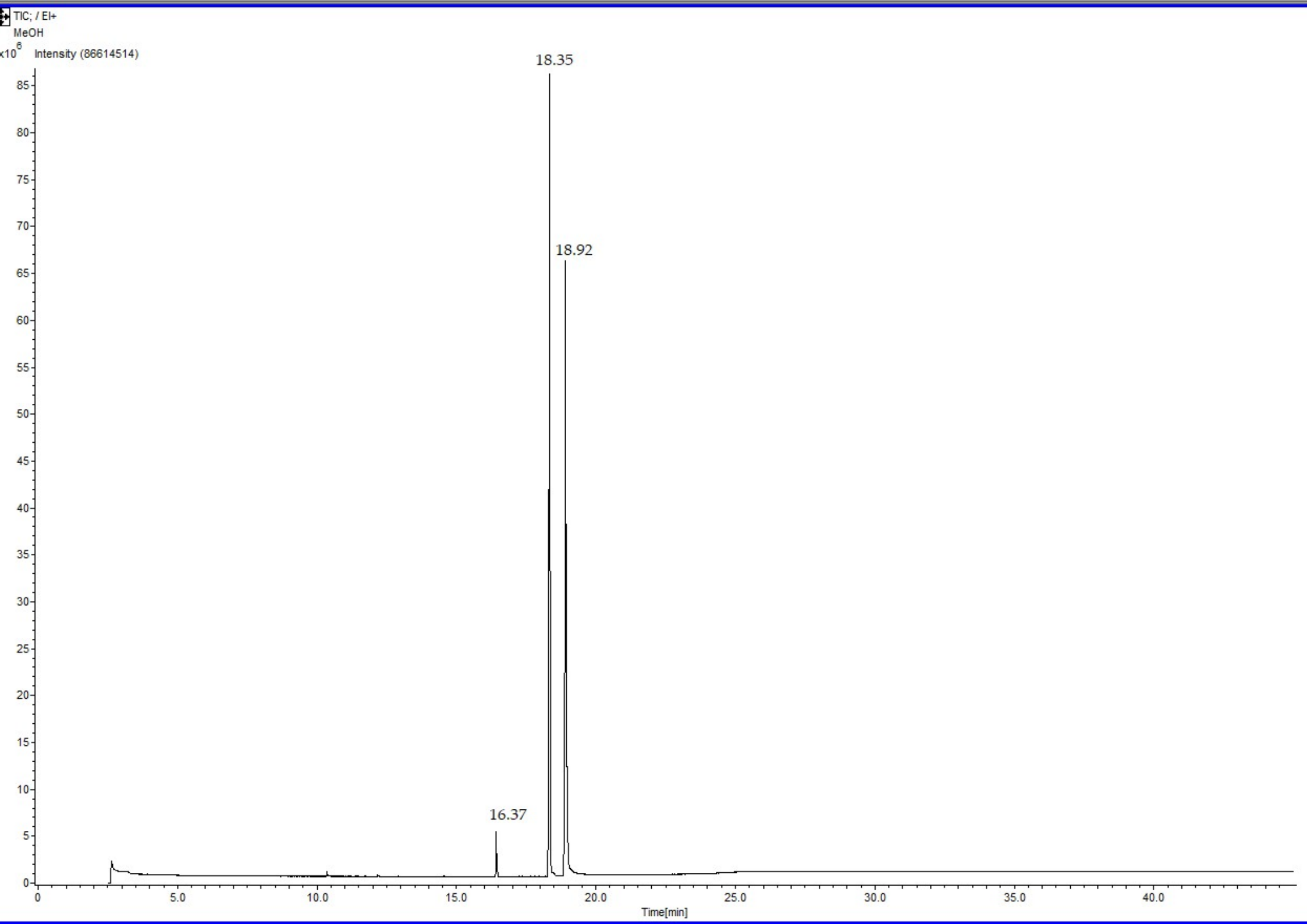

Supplement: Supplementary file 1 [file molecules-24-00889-s001.zip › Figure S9. GC-MS chromatogram of fraction 3.pdf]
